# DART: Distance Assisted Recursive Testing

**Published:** 2023

**Authors:** Xuechan Li, Anthony D. Sung, Jichun Xie

**Affiliations:** Department of Biostatistics, Duke University, Durham, NC 27705, USA; Department of Medicine, Duke University, Durham, NC 27705, USA; Department of Biostatistics, Duke University, Durham, NC 27705, USA

**Keywords:** multiple testing, hierarchical testing, aggregation tree, false discovery rate, auxiliary information

## Abstract

Multiple testing is a commonly used tool in modern data science. Sometimes, the hypotheses are embedded in a space; the distances between the hypotheses reflect their co-null/co-alternative patterns. Properly incorporating the distance information in testing will boost testing power. Hence, we developed a new multiple testing framework named Distance Assisted Recursive Testing (DART). DART features in joint artificial intelligence (AI) and statistics modeling. It has two stages. The first stage uses AI models to construct an aggregation tree that reflects the distance information. The second stage uses statistical models to embed the testing on the tree and control the false discovery rate. Theoretical analysis and numerical experiments demonstrated that DART generates valid, robust, and powerful results. We applied DART to a clinical trial in the allogeneic stem cell transplantation study to identify the gut microbiota whose abundance was impacted by post-transplant care.

## 1 Introduction

Multiple testing is commonly used to discover important features in modern data science. Each feature represents a hypothesis: the important features correspond to alternative hypotheses, and the rest to null hypotheses. A rejected hypothesis corresponds to an identified feature. The goal is to discover the alternative hypotheses with a controlled false discovery rate (FDR), the expected number of false discoveries over the total number of discoveries.

Under many circumstances, the hypotheses are coupled with other attributes in a metric space with a known pairwise distance. For example, previous brain studies have shown that the anatomical distance between the neurons can partially inform brain activities and neurons’ co-functioning patterns (Alexander-Bloch et al., 2013; Perinelli et al., 2019; Kristanto et al., 2020). For another example, the polygenic distance between amplicon sequence variants (ASVs) often informs their functional similarity and survival (Chen et al., 2012; Garcia et al., 2014; Martiny et al., 2015). In these examples, if we form neurons or ASVs as hypotheses, properly incorporating anatomy distance or the polygenic distance between hypotheses can improve the power in identifying functionally important neurons or ASVs. In other words, the distance among the hypotheses partially reflects the hypotheses’ co-null or co-alternative status, called co-status hereafter.

We developed a new hierarchical multiple testing procedure called DART. It incorporates distance information to boost the power of testing. DART has two stages. The first stage is based on AI modeling: we use the automatic algorithm to construct an aggregation tree that incorporates the distance information and facilitates downstream testing. The second stage is based on statistical modeling: we perform a bottom-up multiple testing procedure on the aggregation tree to control FDR. Unlike traditional multiple testing that only uses statistical modeling, DART combines the power of statistical models and AI models to improve testing accuracy: statistical models are less flexible and less data-adaptive, but they can provide highly interpretable results; AI models fail to provide interpretable results, but they can explore complex underlying structures. Our study provides a new solution to generate data-adaptive and highly-interpretable results.

Our work is closely related to three streams of research.

- *Testing using distance/side information*. Some methods incorporate side information via parametric modeling. For example, Shu et al. (2015) imposes a 3D hidden Ising model to model the nearby hypothesis co-status. Lee and Lee (2016) uses a scalar parameter in a specific exponential-family distribution to control the level of co-status among nearby hypotheses. Cai et al. (2020) uses kernel functions to enforce similar prior null probabilities on nearby hypotheses. Lei and Fithian (2018) proposes an iterative FDR control procedure that incorporates side information via a parametric model estimated by the EM algorithm. These methods use parametric forms to model how distance/side information impacts the hypothesis co-status. Although parametric methods usually achieve good performances on simulated data, their performance on real data is unclear. For example, for fMRI analysis, Eklund et al. (2012) used extensive real data sets to show that many commonly used parametric models that specify temporal correlations in fMRI analysis are inappropriate, and thus lead to highly inflated type I error in multiple testing. Recently, Ramdas et al. (2019b) develops an FDR control procedure that allows scientists to incorporate four types of prior knowledge simultaneously. The procedure allows mix and match techniques and using multiple different forms of prior knowledge simultaneously while maintaining internal consistency among the pattern of rejections and acceptances. Besides, Xia et al. (2017) and Tansey et al. (2018) employ neural networks to leverage side information to improve testing accuracy while controlling FDR. All these methods specify the side information for each hypothesis specifically. However, the distance information is not defined for each hypothesis but for each hypothesis pair, and thus cannot be incorporated directly. Alternatively, some methods adopt non-parametric models to incorporate distance information into multiple testing. Zhang et al. (2011) uses a local neighborhood size and uses the aggregated P-values in the neighborhood to perform multiple testing, called ${\text{FDR}}_{L}$. Li et al. (2013) uses the non-parametric propagation-separation approach (Belomestny and Spokoiny, 2007) to smooth the coefficients in the generating generalized estimating equations (GEE) models. These methods are more data adaptive. Because Li et al. (2013) only works for the GEE models, we will mainly compare DART with ${\text{FDR}}_{L}$ later.
- Hierarchical multiple testing.
  - *Gatekeeping*. Suppose the hypotheses are grouped into m≥2 ordered families. Gatekeeping procedures test a later family only if they reject the previous families (Dmitrienko et al., 2007, 2008, 2011; Dmitrienko and Tamhane, 2013; Xi and Tamhane, 2014). The aim is to control the family-wise error rate (FWER). Gatekeeping procedures are usually designed for clinical trials. For other applications, the tests lose power when they discard the families of hypotheses whose previous family is accepted.
  - *Top-down hierarchical testing*. Soriano and Ma (2017) applied a tree-structured Markov prior distribution to the indicators of hypotheses status and then calculated their posterior being alternative. The method relies on parametric modeling. Yekutieli (2008) proposes a top-down testing rule similar to gatekeeping. A node on the tree (a set with multiple hypotheses) will be tested only if its parent node is rejected. Other top-down multiple testing procedure also have been proposed for hypotheses structured in DAG, such as Ramdas et al. (2019a),Loper et al. (2022), Meijer and Goeman (2015), and Goeman and Finos (2012). These methods require the hypotheses to be partially ordered in the DAG, and thus not applicable to general hypotheses testing. Lei et al. (2020) proposes an iterative testing algorithm to perform FDR control on a series of contiguous candidate sets in a constrained set. However, for hypotheses with distance information, how to turn the distance into contiguous candidate sets is unclear.
  - *Bottom-up hierarchical testing*. This approach tests the individual hypotheses first and then tests the aggregated hypotheses later. Our method, DART, adopts this approach. The most similar work to ours is the one introduced by Li et al. (2020b). They proposed a bottom-up procedure to test conjugate nodes (sets of hypotheses) with tree structures. A conjugate node is alternative only when all its containing hypotheses are alternative. Their method aims to control the node-level FDR. In contrast, DART focuses on each hypothesis. It aims to control hypothesis-level FDR. Thus, DART is fundamentally different.
- *Explainable AI (XAI)*. XAI aims to (a) produce more explainable models while maintaining a high level of learning performance (prediction accuracy) and (b) enable human users to understand, appropriately trust, and effectively manage the emerging generation of AI partners. (Turek, 2021) However, to the best of our knowledge, no XAI method has been proposed to control FDR in multiple testing. In general, pure AI modeling introduces intrinsic difficulties in controlling FDR.

## 2 Preliminaries

Suppose there are m hypotheses, forming the null and alternative hypothesis sets $\Omega_{0}$ and $\Omega_{1}:\Omega_{0}\cap \Omega_{1}=\emptyset ,\Omega_{0}\cup \Omega_{1}=[m]$. For hypothesis i, a P-value statistic $T_{i}$ is derived. Previous studies often assume the null P-values follow super-uniform distributions:

$$\forall i\in \Omega_{0},\forall p\in (0,\;1),\text{P}\left(T_{i}\leq p\right)\leq p.$$

Our work relaxes this assumption. We allow a null $T_{i}$ asymptotically converges to a super-uniform statistic ${\tilde{T}}_{i}$ in the following way.

(1) $$\underset{i\in \Omega_{0}}{\text{sup}}\;\underset{p\in {𝒫}_{i0}}{\text{sup}}\;\left|\frac{\text{P}\left(T_{i}<p\right)}{\text{P}\left({\tilde{T}}_{i}<p\right)}-1\right|\leq \delta_{0m},\;\text{where}\underset{m\to \infty }{\text{lim}}\;\delta_{0m}=o(1)\;\;\text{and}\;{𝒫}_{i0}=\left\{p\in [0,\;1]:P\left({\tilde{T}}_{i}<p\right)\geq {\left\{m(\text{log}\;m\;\text{log}\;\text{log}\;m{)}^{1/2}\right\}}^{-1}\right\}.$$

${𝒫}_{i0}$ excludes the left tail regions (close to zero) to make the convergence easier. Otherwise, if $P\left({\tilde{T}}_{i}<p\right)$ is too small, the convergence is hard to achieve. If ${\tilde{T}}_{i}$ follows a super-uniform (resp. uniform) distribution, we call $T_{i}$ asymptotically super-uniform (resp. uniform). We relax the P-value null distribution assumptions because many P-values derived from asymptotic tests (*e.g*., Wald, score, and likelihood-ratio tests) do not follow super-uniform distributions, but they are asymptotically super-uniform.

We do not make assumptions on alternative P-value distributions. Although they can be arbitrary, it is useful to think of the alternative P-values have larger probabilities to be small than the uniform distributions. Thus, many methods reject the hypotheses when the P-values are small. For example, a commonly used P-value threshold (Liu et al., 2013; Cai and Liu, 2016a; Xie and Li, 2018) is

(2) $$\overset{ˆ}{t}=\text{sup}\left\{\alpha_{m}\leq t\leq \alpha :\frac{mt}{\sum_{i\in [m]}\;\;I\left(T_{i}<t\right)}\leq \alpha \right\},\;\text{where}\;\alpha_{m}=(m\;\text{log}\;m{)}^{-1}.$$

Here, the dotted fraction notation denotes the shorthand $\overset{a}{\ddot{b}}=\frac{a}{b\vee 1}$. The threshold $\overset{ˆ}{t}$ for the Benjamini and Hochberg procedure (BH) (Benjamini and Hochberg, 1995) is slightly different but asymptotically equivalent to (2). In general, the rejection set is $ℛ=\left\{i:T_{i}\leq t\right\}$ for some threshold t. Then, $𝒰=\Omega_{1}\cap ℛ$ is called the true discovery set, and $𝒱=\Omega_{0}\cap ℛ$ is called the false discovery set. The false discovery proportion and rate are defined as

$$\text{FDP}≔\frac{\left|𝒱\right|}{\left|ℛ\right|},\;\text{FDR}\;≔\text{E}\left(\text{FDP}\right),$$

where |𝒜| denotes the cardinality of set 𝒜. Our task is to design a powerful rejection rule to asymptotically control FDR when m goes to infinity. We hope to gain power by properly incorporating the distance information between hypotheses, assuming they reflect their co-status patterns.

## 3 DART Algorithm Description

DART has two stages. Stage I uses an AI method to transfer the distance matrix into an aggregation tree, which defines the testing structure (Section 3.1). Stage II tests hypotheses embedded in the tree to gain power while controlling FDR (Section 3.2). An illustrating example is provided in Section 3.3.

### 3.1 Distance Matrix and Aggregation tree

Stage I aims to construct an aggregation tree 𝒯 that provides a hierarchical testing structure for stage II. The aggregation tree 𝒯 has L layers, constructed based on the distance matrix $D=\left(d_{ij}\right)$. The node-set on layer ℓ is denoted by ${𝒜}^{\left(\ell \right)}$. On the first (bottom) layer, ${𝒜}^{\left(1\right)}=\{\{1\},\ldots ,\{m\}\}$; each node represents a hypothesis. In general, ${𝒜}^{\left(\ell \right)}$ contains the nodes representing a set of hypotheses.

The hypotheses have different co-status patterns: some alternative hypotheses might stand-alone, some co-alternative within a small region, and others co-alternative within a large region. For any node A, denote its diameter (within-node distance) by $\text{dia}\;(A)={\text{max}}_{i,j\in A}\;d_{ij}$. On higher layers of 𝒯, nodes have larger diameters. We design multiple layers to construct nodes with various diameters and adapt to various co-status patterns.

In stage II, if a node is rejected, we will reject all the hypotheses within the node. For this purpose, we require the hypothesis distance within the node to be small so that they are likely co-status; thus, imposing the same decision rule on these hypotheses is reasonable. We require that for all $A\in {𝒜}^{\left(\ell \right)},\;\text{dia}\;(A)\leq g^{\left(\ell \right)}$, where $g^{\left(\ell \right)}$ is a distance threshold with $0<g^{\left(2\right)}<\ldots <g^{\left(L\right)}$. We set $g^{\left(1\right)}=0$ because we do not need to aggregate hypotheses on the first layer.

Another requirement is to limit nodes’ child numbers. Any node A in ${𝒜}^{\left(\ell \right)}$ with ℓ≥2 is formed by aggregating the nodes from the previous layer. These nodes are called the children of A; they form the child set 𝒞(A). We require that |𝒞(A)|≤M for any node A. Here, M is called the maximum node size. We set up this requirement to increase the following testing’s stability: if A contains too many hypotheses, rejecting A leads to rejecting all hypotheses in A. This introduces large variability in testing and makes the algorithm outputs unstable.

To make sure all the nodes on layer ℓ satisfy $\text{dia}\;(A)\leq g^{\left(\ell \right)}$ and |𝒞(A)|≤M, we developed Algorithm 1. The key strategy is to recursively set ${𝒜}^{\left(\ell \right)}$ based on ${𝒜}^{\left(\ell -1\right)}$ and find the closest node pair for aggregation. The resulting tree depends on the tuning parameter L,M, and $g^{\left(\ell \right)}$ with ℓ∈{2,…,L}. We introduce the tuning parameter selection criteria in Section 3.4.

Algorithm 1 is a feasible algorithm to construct an aggregation tree satisfying $\text{dia}\;(A)\leq g^{\left(\ell \right)}$ and |𝒞(A)|≤M. Alternative AI approaches could also be used. For example, an aggregation tree can be constructed by recursively applying community detection algorithms, such as K-means clustering (Jin and Han, 2010), Louvain method (Blondel et al., 2008), and Leiden algorithm (Traag et al., 2019), in the similar spirit of hierarchical clustering (Zepeda-Mendoza and Resendis-Antonio, 2013). If a modified algorithm based on these algorithms could restrict the maximum node size, it may also be applied here. The key of the desired algorithm is to generate a tree with few mixed nodes (defined in Section 4) to ensure the asymptotic FDR control of Step 2.

### 3.2 Recursive Testing Embedded in the Tree

Recall that the tree nodes consist of close hypotheses likely to be co-status. We developed a multiple testing algorithm that incorporates the tree to improve the testing power.

Algorithm 2 describes the recursive testing procedure from the single-hypothesis layer (bottom) to the large-node layer (top). It starts with testing all hypotheses using the traditional FDR control procedure. This step does not use any hypothesis co-status patterns. Thus, DART is likely to discover the hypotheses with strong signal-to-noise ratios (SNRs) on the bottom layer. On higher layers, DART tests larger nodes containing more hypotheses. The weaker-SNR hypotheses are likely to be aggregated then to increase their identification chances.

Algorithm 2 mentioned a few new terms: dynamic nodes, candidate node P-values, and recursive P-value cutoffs. We provide more details on these terms below.

#### Candidate dynamic nodes.

The dynamic nodes are the nodes excluding the rejected hypotheses on previous layers. If a hypothesis is rejected, we will not test it again. There are two main reasons. First, if the rejected hypotheses were not removed, they could become non-significant after being aggregated with other null hypotheses on higher layers. It introduces interpretation difficulties. Second, a rejected hypothesis on low layers usually has strong SNRs. If we include these hypotheses in higher layers, this hypothesis alone may make the entire node significant, even if the node contains null hypotheses. Then, the rejection of the entire node may lead to false rejections on its containing null hypotheses.

A candidate dynamic node is a dynamic node with |𝒞(S)|≥2. If a node S has |𝒞(S)|=1, this node must have been tested on previous layers. Thus, we do not need to test it again. The set of the candidate dynamic nodes on layer ℓ is denoted by ${ℬ}^{\left(\ell \right)}$.

#### Candidate node P-values.

For any candidate dynamic node S, we use the Gaussian aggregation approach to derive candidate node P-value: $T_{S}=\overset{‾}{\Phi }\left\{\sum_{j\in S}\;{\overset{‾}{\Phi }}^{-1}\left(T_{j}\right)/\sqrt{\left|S\right|}\right\}$, where $\overset{‾}{\Phi }$ is the complement cumulative density function (CCDF) of the standard Gaussian distribution. The aggregation is efficient. Alternatively, we may consider using other aggregation approaches, such as the Fisher’s combination (Fisher, 1925), the chi-square aggregation, and the Cauchy aggregation (Liu and Xie, 2020). The Fisher’s combination and the chi-square aggregation approaches have lower power than the Gaussian aggregation when the hypotheses in the node are co-status. The Cauchy aggregation relies on the heavy-tail Cauchy density; thus, it introduces theoretical challenges to studying the asymptotic null distributions of the node P-values. The main challenge lies in accounting for the post-selection effect: the hypotheses and their P-values only have the chance to be aggregated when they are not rejected on the previous layers. In contrast, for Gaussian aggregation, the post-selection problem can be solved because the candidate node P-values are still asymptotically super-uniform (Lemmas 7 and 8). Thus, we go with the Gaussian aggregation.

#### P-value cutoffs.

On layer ℓ, we already have the set of the rejected hypotheses on the previous ℓ-1 layers, denoted by ${ℛ}^{\left(1:\ell -1\right)}$. We set up the P-value threshold ${\overset{ˆ}{t}}^{\left(\ell \right)}$ as

(3) $${\hat{t}}^{\left(\ell \right)}=\text{sup}\left\{\alpha_{m}\leq t\leq \alpha :\frac{\sum_{\ell^{\prime }=1}^{\ell -1}m^{\left(\ell^{\prime }\right)}{\hat{t}}^{\left(\ell^{\prime }\right)}+m^{\left(\ell \right)}t}{\left|{ℛ}^{\left(1:\ell -1\right)}\right|+\underset{S\in {ℬ}^{\left(\ell \right)}}{\sum }\left|S\right|I\left(T_{S}<t\right)}\leq \alpha \right\}.$$

Here, $\alpha_{m}=(m\;\text{log}\;m{)}^{-1}$ and $m^{\left(\ell^{\prime }\right)}=\left|{ℬ}^{\left(\ell^{\prime }\right)}\right|$. It is easy to see that ${\overset{ˆ}{t}}^{\left(\ell \right)}$ depends on the previous layer’s cutoff and rejection set. Comparing $\overset{ˆ}{t}$ in (2) and ${\overset{ˆ}{t}}^{\left(\ell \right)}$ in (3), it is easy to see that they share some similarities. The numerators bound the false discoveries asymptotically, and the denominators count the total discoveries. By making the fraction less than or equal to the desired FDR level α, DART asymptotically controls the weighted node-FDR (Section 4). DART also asymptotically controls the hypothesis level FDR when most nodes contain co-status hypotheses.


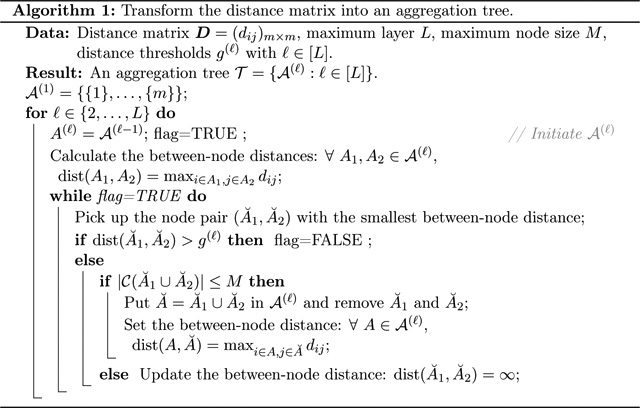



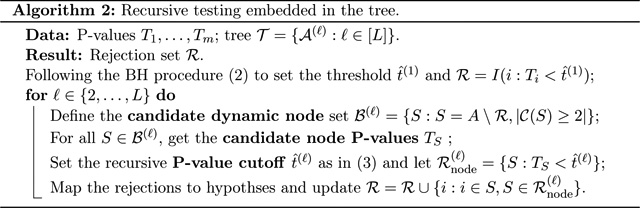


### 3.3 A Toy Example to Illustrate DART

**Figure 1:**
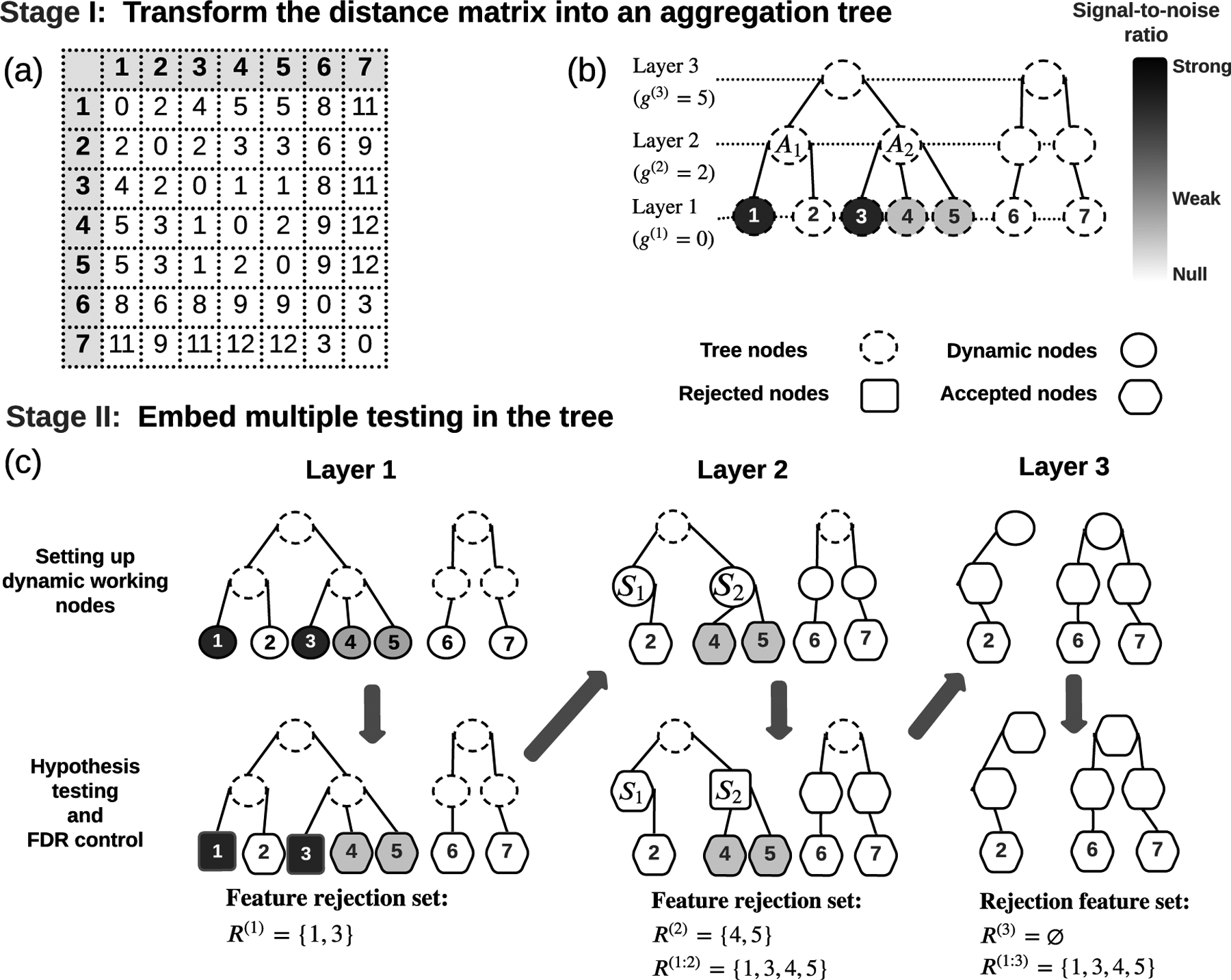
An illustrating example of DART with 7 features.

We provide a toy example in Figure 1 to illustrate DART. The detailed algorithm descriptions are provided in Algorithm 1 and Algorithm 2 in Section 3.

Figure 1(a) shows the distance matrix of the seven hypotheses. In stage I, we transfer the distance matrix into the 3-layer aggregation tree based on Algorithm 1. The resulting 3-layer aggregation tree is shown in Figure 1(b). Each leaf (node on the bottom layer) on the tree corresponds to a hypothesis and a test statistic. The gray scales illustrate the underlying SNR ratios in the statistics; these SNR ratios are unknown.

In stage II, we perform the multiple testing embedded in the aggregation tree based on Algorithm 2. We start from the bottom layer and hierarchically proceed to higher layers.

- On layer 1 (the bottom layer), hypotheses 1 and 3 are rejected because their test statistics have strong SNR ratios.
- After the testing procedure on each layer, the rejected nodes are marked by solid squares and the accepted nodes solid hexagons. If a node is rejected, all its containing hypotheses are rejected.
- When testing on a higher layer, all previously rejected features are excluded from the nodes (dashed-line circled) to form the dynamic nodes (solid-line circled) on this layer. For example, on layer 2, tree node $A_{1}=\{1,\;2\}$ turns into the dynamic node $S_{1}=\{2\}$ because hypothesis 1 is rejected on the bottom layer. $S_{1}$ will not be tested on layer two because it only contains hypothesis 2, already tested on layer 1. Using the terminology in Section 3.2, the number of children $\left|𝒞\left(S_{2}\right)\right|=1$; thus, $S_{1}$ is not a candidate dynamic node and will not be tested on layer 2. For another example, $A_{2}=\{3,\;4,\;5\}$ turns to the dynamic node $S_{2}=\{4,\;5\}$ on layer 2 because hypothesis 3 was rejected on the bottom layer. $\left|𝒞\left(S_{2}\right)\right|=2$ and thus, it will be tested on layer 2.
- The test statistics of hypotheses 4 and 5 have relatively weak SNRs. They are not significant enough to be rejected on layer 1. However, on layer 2, they are aggregated to form the dynamic node $S_{2}=\{4,\;5\}$; the aggregated SNR is large enough so that $S_{2}$ is rejected, leading to the rejections of hypotheses 4 and 5. Thus, the power of DART is higher than the power of a single-layer testing method.

### 3.4 Tuning Parameter Selection

Proper parameters will result in a tree empowering testing. We suggest setting the maximum node size M as 2 or 3. Appendix C verified that when M=2 or M=3, DART asymptotically controls FDR and is more powerful. Denote the desired minimal number of nodes on layer L by $c_{m}$. If $c_{m}<35$, DART’s asymptotic validity might fail to kick in, leading to possibly inflated FDR. Therefore, we request $c_{m}\geq 35$. Accordingly, we set the maximum layer number $L=\left\lfloor {\text{log}}_{M}\;m-{\text{log}}_{M}\;c_{m}\right\rfloor \leq \left\lfloor {\text{log}}_{M}\;m-{\text{log}}_{M}\;35\right\rfloor$. The distance thresholds are set recursively to maximize the number of candidate nodes on each layer. We first try a set of possible thresholds $G=\left\{g_{1},\ldots ,g_{K}\right\}$. On layer ℓ with ℓ≥2, we let $G^{\left(\ell \right)}=\left\{g\in G:g\geq g^{\left(\ell -1\right)}\right\}$. Hierarchically, on layer ℓ, we try every $g\in G^{\left(\ell \right)}$ and count the number of resulting candidate nodes on this layer. We set $g^{\left(\ell \right)}$ as the g with the most candidate nodes. See Algorithm 3 in Appendix C for details.

## 4 Asymptotic Validity

This section shows that DART asymptotically controls the hypothesis FDR under mild conditions.

### Weighted node-FDR and hypothesis FDR.

For any candidate dynamic node, if the node contains any alternative hypothesis, we call the node alternative; otherwise, it is called null. On layer ℓ, we denote the set of null candidate dynamic nodes by ${ℬ}_{0}^{\left(\ell \right)}$ and the set of rejected nodes by ${ℛ}_{\text{node}}^{\left(\ell \right)}$. Then the weighted node-FDR is

$${\text{FDP}}_{\text{node}}≔\frac{\sum_{\ell =1}^{L}\sum_{S\in {ℛ}_{\text{node}}^{\left(\ell \right)}\cap {ℬ}_{0}^{\left(\ell \right)}}\left|S\right|}{\sum_{\ell =1}^{L}\sum_{S\in {ℛ}_{\text{node}}^{\left(\ell \right)}}\left|S\right|}=\frac{\sum_{\ell =1}^{L}\sum_{S\in {ℛ}_{\text{node}}^{\left(\ell \right)}\cap {ℬ}_{0}^{\left(\ell \right)}}\left|S\right|}{\left|ℛ\right|},\;{\text{FDR}}_{\text{node}}=\text{E}\left({\text{FDP}}_{\text{node}}\right).$$

In contrast, the hypothesis FDR is

$$\text{FDP}≔\frac{|ℛ|\cap \Omega_{0}}{\left|ℛ\right|},\;\text{FDR}=\text{E}\left(\text{FDP}\right).$$

Notably, $\;{\text{FDP}}_{\text{node}}$ only accounts for the false discoveries in null nodes. If an alternative node containing both null and alternative hypotheses is rejected, the rejection will not increase the numerator of ${\text{FDP}}_{\text{node}}$ but will increase the numerator of FDP. Thus, ${\text{FDP}}_{\text{node}}\leq \text{FDP}$. Although our ultimate goal is to control FDP, we control ${\text{FDP}}_{\text{node}}$ as an intermediate step. The difference between ${\text{FDP}}_{\text{node}}$ and FDP relies on the number of the rejected mixed nodes that contain both null and alternative hypotheses.

### Weighted node-FDR control.

We require the following conditions for weighted node-FDR control.

- *Condition 1. Sparse alternatives*. The alternative hypothesis number $m_{1}=O\left(m^{r_{1}}\right)$, for some $r_{1}<{\left(M^{L-1}+1\right)}^{-1}$.
- *Condition 2. Sufficient moderate SNR nodes (see Definition 4 in* Appendix A). Denote the number of moderate SNR nodes on the tree 𝒯 by $m_{\text{md}}$. We require that $m_{\text{md}}\geq O(\text{log}\;m)$. A moderate SNR node (a) contains no hypotheses that will be rejected with non-vanishing probabilities before its locating layer, and (b) will be rejected on its locating layer with a non-vanishing positive probability. The existence of these nodes is to guarantee that some alternative nodes are rejected on each layer so that the threshold ${\overset{ˆ}{t}}^{\left(\ell \right)}$ is not too small; otherwise, the number of total rejections will be too small so that a single false rejection would inflate FDR.
- *Condition 3. Almost independence*. Most hypothesis P-values are mutually independent. The number of dependent P-values does not exceed $o\left(m_{\text{md}}\right)$.

Here, Conditions 1 and 2 are inherited and extended from the previous multiple testing literature (Cai and Liu, 2016b). Condition 1 assumes the alternative hypothesis sparsity. Condition 2 usually holds when the sample size n is sufficiently large compared to p,L is properly chosen, and the signal-to-noise ratio distribution of the alternative hypotheses has continuous support over a large range. Condition 3 is a strong assumption. We require it to ensure that after higher-layer aggregation, most node P-values are still asymptotically super-uniformly distributed under the null. It is possible to relax this condition. However, the proof will be much more complicated.

**Lemma 1** *Under Conditions 1–3, at any pre-specified level* α∈(0,1), Algorithm 2 *satisfies the following asymptotic validity*.

- *For any* $\epsilon >0,{\text{lim}}_{m\to \infty }\;\text{P}\left(FDP_{\text{node}}\leq \alpha +\epsilon \right)=1$. *Consequently, ${\text{lim}}_{m\to \infty }\;FDR_{\text{node}}\leq \alpha$*.
- *Let* ${\tilde{\Omega }}_{0}$ *be the set of null P-values that are asymptotically uniform. If* ${\text{lim}}_{m\to \infty }\;\left|{\tilde{\Omega }}_{0}\right|/m=1$, *then for any* $\epsilon >0,{\text{lim}}_{m\to \infty }\;\text{P}\left(\left|FDP_{\text{node}}-\alpha \right|\leq \epsilon \right)=1$. *Consequently*, ${\text{lim}}_{m\to \infty }\;FDR_{\text{node}}=\alpha$.

See Appendix B for proof of this lemma. Two main challenges in the proof are the hierarchical testing structure and the post-selective effect introduced by the dynamic nodes. Thus, we proved the ${\text{FDR}}_{\text{node}}$ control recursively, starting from the bottom layer. The bottom layer follows the BH procedure. Then, given the ${\text{FDR}}_{\text{node}}$ control on the previous layers, we proved the control on the current layer. Recall that dynamic nodes do not contain the already-rejected hypotheses. To account for the post-selection effect, we proved that conditioning on the testing results from the previous layers, the dynamic node P-values are still asymptotically super-uniform or asymptotically uniform.

### Hypothesis FDR control.

Previously, we constructed a tree where the hypotheses were hierarchically aggregated into the nodes based on their distance. Thus, we expect many nodes contain co-status hypotheses. However, some large nodes on high layers may be mixed, containing null and alternative hypotheses. Some mixed nodes are concerning, while others are not. For example, suppose a node A on layer ℓ contains null hypotheses, strong SNR hypotheses (see Definition 5 in Appendix A) and weak SNR hypotheses (see Definition 6 in Appendix A). The strong SNR hypotheses are probably rejected before layer ℓ. Thus, when Algorithm 2 reaches layer ℓ,A probably already turns into a dynamic node only containing the null and weak SNR hypotheses. The weak SNR hypotheses are the alternative hypotheses unlikely to be rejected. As a result, the null hypotheses in A will not be rejected, and thus, the existence of A will not inflate the FDR. Thus, to control FDR, we only need to restrict those concerning mixed nodes whose null hypotheses are likely to be rejected with non-vanishing probabilities.

**Definition 2 (Concerning mixed nodes)** *For any node* $A\in {𝒜}^{\left(L\right)}$, *let*

$$A^{*}=A\setminus \left(\Omega_{st}\cup \Omega_{wk}\right),$$

where $\Omega_{st}$ is the strong *SNR* hypothesis set, and $\Omega_{wk}$ is the weak *SNR* hypothesis set. The definitions of $\Omega_{st}$ and $\Omega_{wk}$ are provided in Appendix A. If $A^{*}\cap \Omega_{0}\neq \emptyset$ and $A^{*}\cap \Omega_{1}\neq \emptyset$, we call A a concerning mixed node.

- *Condition 4*. sparse concerning mixed nodes. On the top layer of the tree ${𝒜}^{\left(L\right)}$, the number of the concerning mixed nodes cannot exceed $o\left(m_{\text{md}}\right)$.

We allow the existence of concerning mixed nodes, but to asymptotically control FDR, Algorithm 2 cannot afford too many of them. Condition 4 specifies the tolerance level. Intrinsically, Condition 4 depends on the assumption that the distance matrix predominantly reflects the hypothesis co-status. If so, with properly selected $\left\{g^{\left(\ell \right)}:\ell \in [L]\right\}$, Algorithm 1 will probably generate a tree satisfying Condition 4, because it uses the greedy algorithm to aggregate the closest remaining hypotheses. On the other hand, if this assumption does not hold, Algorithm 1 cannot generate a tree with nodes implying hypothesis co-status. Under this case, we do not recommend using DART.

By adding Condition 4, we extend the ${\text{FDR}}_{\text{node}}$ control to FDR control (Theorem 3).

**Theorem 3** *Under Conditions 1–4, at any pre-specified level* α∈(0,1), Algorithm 2 *satisfies the following asymptotic vality*.

- *For any* $\epsilon >0,{\text{lim}}_{m\to \infty }\;\text{P}(FDP\leq \alpha +\epsilon )=1$. *Consequently*, ${\text{lim}}_{m\to \infty }\;FDR\leq \alpha$.
- *Let* ${\tilde{\Omega }}_{0}$ *be the set of null P-values that are asymptotically uniform. If ${\text{lim}}_{m\to \infty }\;\left|{\tilde{\Omega }}_{0}\right|/m=1$*, *then for any* $\epsilon >0,{\text{lim}}_{m\to \infty }\;\text{P}(|FDP-\alpha |\leq \epsilon )=1$. *Consequently*, ${\text{lim}}_{m\to \infty }\;FDR=\alpha$.

## 5 Numerical Simulation

We simulated m=1000 features located in the two-dimensional Euclidean space with randomly generated location coordinates: the first coordinate follows N(0,2), and the second coordinate follows Unif(0,4). A distance matrix $D={\left(d_{ij}\right)}_{m\times m}$ was calculated based on the Euclidean distance between two features’ locations. Feature i links to a parameter of interest $\theta_{i}$. The hypotheses are $H_{0,i}:\theta_{i}=0$ versus $H_{1,i}:\theta_{i}\neq 0,i\in [m]$.

We considered four settings, SE1-SE4. SE1 simulated a straightforward case where the P-values follow uniform distributions under the null. SE2 misspecified the null distributions of the test statistics, in order to evaluate the methods’ robustness. SE3 simulated the linear regression model, and SE4 simulated the Cox proportional hazard model; their P-values were derived from the Wald tests. Each setting contained 200 repetitions. The setting details were described in Appendix C.

Under different nominal FDR levels α∈{0.05,0.1,0.15,0.2}, we compared the performance of DART and its competitors: BH (Benjamini and Hochberg, 1995), AdaPT (Lei and Fithian, 2018) and ${\text{FDR}}_{L}\left({\text{FDR}}_{L}\right.$ I and ${\text{FDR}}_{L}$ II) (Zhang et al., 2011). AdaPT incorporates the location coordinates as side information; DART incorporates the distance matrix; ${\text{FDR}}_{L}$ incorporates the information of each hypothesis’s k nearest neighbors. Thus, the settings favor AdaPT because we provided it with the most information. The tuning parameters used in DART, AdaPT, and ${\text{FDR}}_{L}$ procedures were discussed in Appendix C.

**Figure 2:**
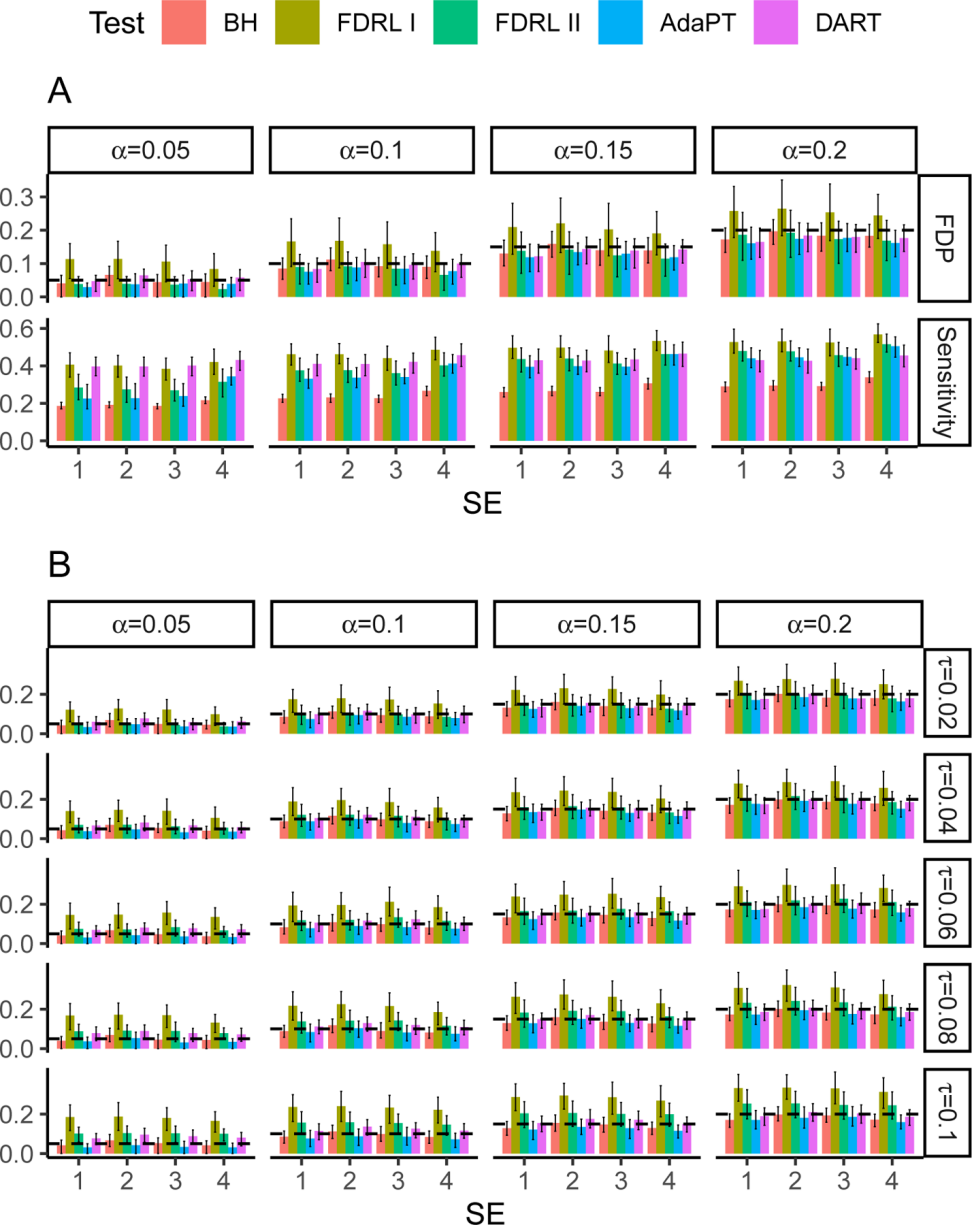
A, FDP and sensitivity of various testing methods under SE1-SE4. B, FDP of various testing methods under SE1-SE4 when proportion τ of the alternative hypotheses were switched with the null. The dashed lines in the FDP panels mark the nominal FDR levels.

Figure 2A shows the type I error (measured by average FDP) and power (measured by average sensitivity) and their error bars of various methods:

- *Average FDP*: Under SE1, SE3, and SE4, DART, BH, AdaPT, and ${\text{FDR}}_{L}$ II control the average FDP well. Under SE2, DART’s (resp. BH’s) average FDP is slightly inflated when α=5% (resp. ≤15%). This is because we deliberately misspecified the P-value null distributions in SE2. AdaPT has consistently good FDR control. In contrast, ${\text{FDR}}_{L}$ I exhibited severe FDR inflation under all four settings. ${\text{FDR}}_{L}$ I has longer error bars than DART; so does ${\text{FDR}}_{L}$ II when α≥10%. This suggests that DART’s FDP is less variable than ${\text{FDR}}_{L}$ I and II.
- *Average sensitivity*: DART’s sensitivities are consistently higher than BH. DART has much higher sensitivities than AdaPT (resp. ${\text{FDR}}_{L}$ II) when α≤15% (resp. α≤10%) and slightly lower sensitivities when α=20%. If a low nominal FDR level (such as 5%) is preferred, DART is the most powerful among all methods.
- *Computation time*: DART is computationally efficient. For example, on average, one run (per repetition) of DART takes only 0.64 minutes across all settings. In contrast, AdaPT failed in generating any testing results within 8 minutes in about 17% of the runs (Table 1 in Appendix C). Among AdaPT’s successful runs (within 8 minutes), one run on average takes 3.90 minutes. DART is at least 6 times faster.

DART assumes that the distances reflect the hypothesis co-status patterns. However, in practice, this assumption could be partially violated. To assess the methods’ robustness, we switched the proportion τ of the alternative hypotheses with the null hypotheses (Appendix C). Figure 2B shows that ${\text{FDR}}_{L}$ have inflated average FDP when the switching proportion τ≥6%. All other methods still have good FDR control. Even under these assumption partial violation cases, DART’s sensitivity is still much higher than BH. Compared to AdaPT, DART still has higher sensitivity when α≤15% and a slightly lower average sensitivity when α=20%. These results show that DART’s performance is consistently satisfying even when the data are less ideal.

One reason for DART’s satisfying performance is because DART incorporates AI modeling to transfer the distance matrix into an aggregation tree, which later defines the testing structure. The AI modeling is data adaptive (compared to fixed neighborhood modeling as in ${\text{FDR}}_{L}$) and robust (compared to the parametric modeling as in AdaPT) and thus generate satisfying results under various settings.

## 6 Real-world Experiment

We applied DART to a clinical trial on hematopoietic stem cell transplantation (HCT). Graft-versus-host disease (GVHD) is one of the major complications of HCT. Recent studies have linked GVHD to the disruptions of the gut microbiome (Jenq et al., 2012). The disruptions may be related to the environmental changes such as post-transplant care (Claesson et al., 2012). This study investigates the impact of two post-transplant cares, home care (HC) and standard hospital care (SH), on patients’ gut microbiota compositions.

In our data, patient fecal samples were collected before and after HCT; the fecal microbiome are sequenced by the 16S ribosomal RNA sequencing at the Memorial Sloan Kettering Cancer Center. The data were then pre-processed by the R package, DADA2 (Callahan et al., 2016), to generate the amplicon sequence variants (ASV) and the read counts. To improve the analysis quality, we removed the ASVs present in fewer than 10% of the samples. Samples with follow-up time longer than 1-year was also removed from the study. The zero counts were replaced by 0.5 (Aitchison, 1982; Kurtz et al., 2015).

After pre-processing, our data contain 456 microbiome samples from 126 leukemia patients before and after the HCT. Each microbiome sample contains 866 amplicon sequence variants (ASVs). We excluded the 9 ASVs with missing taxonomy order information. In microbiome studies, the ASV relative abundance (measured by its abundance proportions) is more meaningful than its absolute abundance. Thus, for the remaining 857 ASVs, we calculated their log odds. Here, the odds for an ASV is

$$\text{odds}=\frac{\text{ASV}\;\text{abundance}\;\text{proportion}}{1-\text{ASV}\;\text{abundance}\;\text{proportion}}.$$

These ASV’s abundance proportions do not add up to 1 because 9 ASVs were excluded. We set up the longitudinal linear mixed model

(4) $${\text{Y}}_{ijk}=\theta_{0,i}+\theta_{1,i}W_{1,k}+\theta_{2,i}W_{2,jk}+\theta_{3,i}W_{1,jk}W_{2,jk}+b_{ij}+\epsilon_{ijk}.$$

Here i is the ASV index, j is the sample index, and k is the patient index. The outcome ${\text{Y}}_{ijk}$ is the log odds of ${\text{ASV}}_{i}$ when sample j of patient k was collected. For patient $k,W_{1,k}$ is one’s after-transplant care type (HC for 1 and SH for 0), $W_{2,jk}$ is care time length. $b_{ij}$ is the random effect to incorporate the dependence across measurements for the same patient, and $\epsilon_{ijk}$ is the random error. To identify ASVs whose abundance change is impacted by the after-transplant care (the interaction between the post-transplant care type and time), we set up the hypotheses: $H_{0,i}:\theta_{3,i}=0,i\in [857]$. The distances between the hypotheses were defined by the evolutionary distances among ASVs. Previous studies showed that evolutionally close ASVs might be functional similar. (Chen et al., 2012; Garcia et al., 2014; Martiny et al., 2015). We used the Wald tests to calculate the P-values.

**Figure 3:**
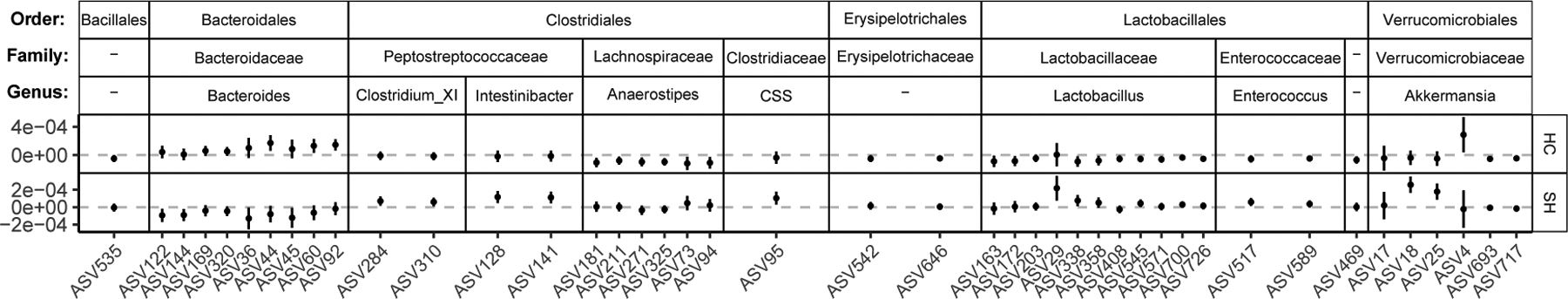
Forest plots to visualize the after-transplant time effect in home care group (HC) and standard hospital care group (SH) on the abundance of 43 DART-identified ASVs. A dot represents the estimated time effect on an ASV; a vertical line marks its 95% confidence interval. Here, CSS stands for Genus Clostridium sensu stricto.

We applied BH and DART to test the hypotheses with the nominal FDR level 5%. Details about aggregation tree construction in DART can be found in Appendix C. BH failed to identify any important ASVs. In contrast, DART identified 43 ASVs by incorporating the evolutionary distance information among ASVs. Among them, 39 ASVs have well-annotated Genus information. Figure 3 shows their log relative abundance change across time in home-care (HC) and standard hospital care (SH) groups Higher abundance in *Enterococcus, Clostridium XI* and *Akkermansia* were associated with more severe GVHD (Payen et al., 2020; Li et al., 2020a a; Shono et al., 2016). Most of the ASVs in these three genera had less abundance over time in the home care group, suggesting home care might help reduce the GVHD severity. Higher abundances in *Bacteroides, Anaerostipes and Lactobacillus* were associated with reduced GVHD severity (Payen et al., 2020; Lin et al., 2021). Almost all the identified ASVs in *Bacteroides* had increased relative abundance in the home care group but decreasing abundance in the standard hospital care group. These findings suggested that home care might help relieve the GVHD severity.

## 7 Conclusion and Discussion

In this paper, we developed a novel multiple testing method, DART, to incorporate feature distance in multiple testing. Under many application contexts, the feature distances serve as auxiliary information of their co-importance pattern. DART incorporates this information to boost the testing power. DART applies to the P-values obtained from many asymptotic tests, and thus can work with a wide range of models.

Stage 1 of DART involves constructing an aggregation tree. We provided Algorithm 1 to construct the aggregation tree. Other algorithms may also work, and result in a different aggregation tree from the same distance matrix. Consequently, Stage 2 testing process could lead to different results based on different trees. In practice, if several aggregation trees exist, DART can be applied to all of them, and we can take the one with the most rejections. The asymptotic validity will still hold for this procedure.

The main limitation of the work is that the FDR control is asymptotic and relies on several conditions. Recently, many hypothesis testing literature develops finite-sample FDR control procedures. These procedures usually impose stronger assumptions on p-values or test-statistics (Lei and Fithian, 2018; Ren and Candès, 2020). The primary obstacle for incorporating these conditions into DART lies in ensuring that higher layer p-values or statistics also adhere to them, thereby facilitating higher layer FDR control proof via deduction. This intriguing area of research warrants further exploration. Additionally, we aspire to alleviate Condition 4 without relying on the presumption that the distance matrix primarily represents co-status patterns. Our objective is to devise a robust testing algorithm that ensures FDR control in the absence of this assumption while enhancing power when the assumption is valid.

In conclusion, our paper initiates an attempt at joint AI-statistics modeling to generate data-adaptive, powerful, and high-interpretable analysis results. It can be easily extended to the case where other information implies the co-importance pattern of the features. Such information could from domain knowledge, external data sets, or other resources. In addition, the hierarchical testing ideas and techniques can also be extended to solve other multiple testing problems.

## Supplementary Material

1

## Figures and Tables

**Figure 4:**
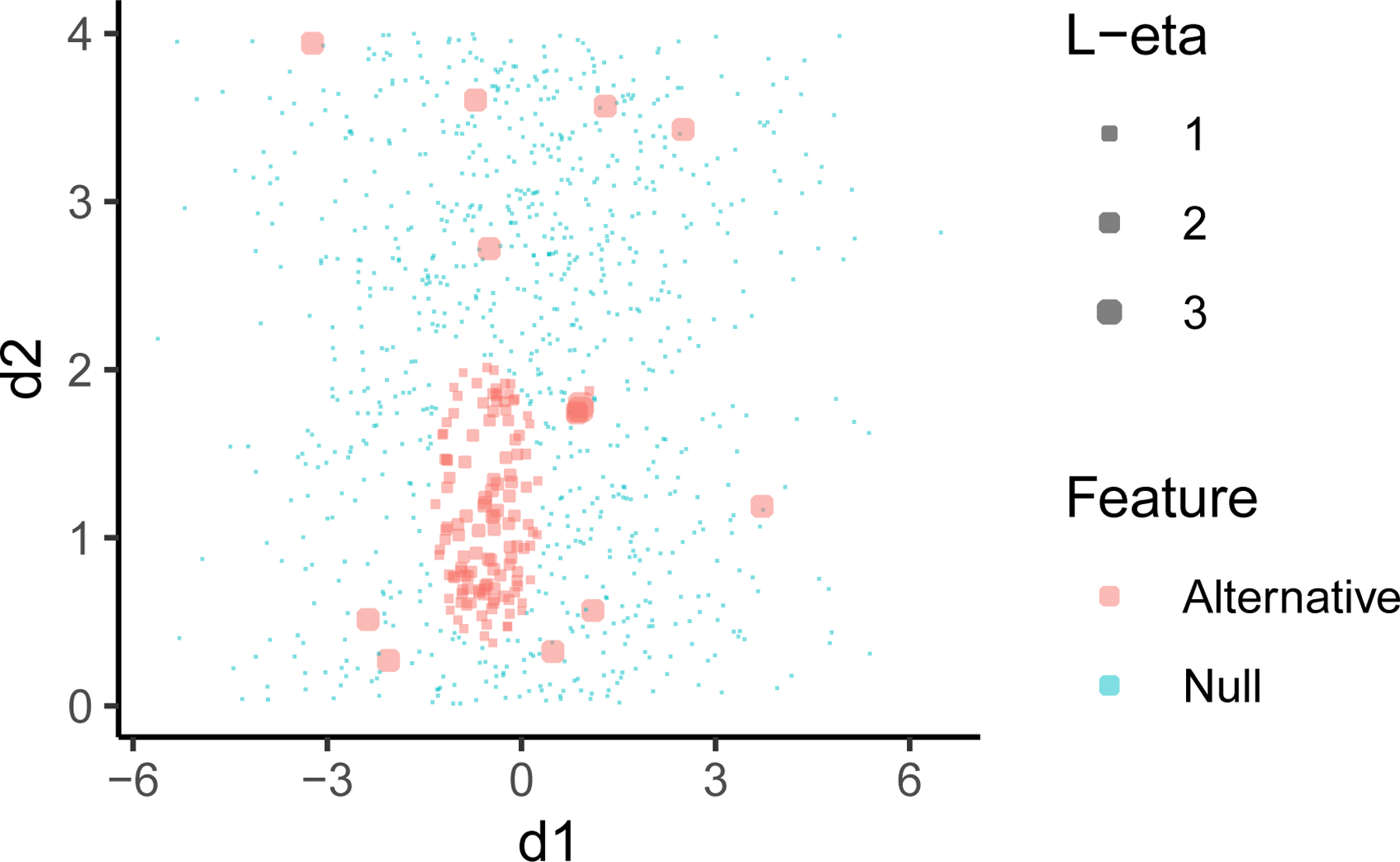
Illustration of the simulated hypotheses’ affiliated location and their corresponding $\eta_{i}$. A dot stands for a hypothesis. The dot color indicates its corresponding hypothesis status; and its size is $\text{L}-\text{eta}=\text{log}\;\left(\eta_{i}+1\right)+0.01$.

**Figure 5:**
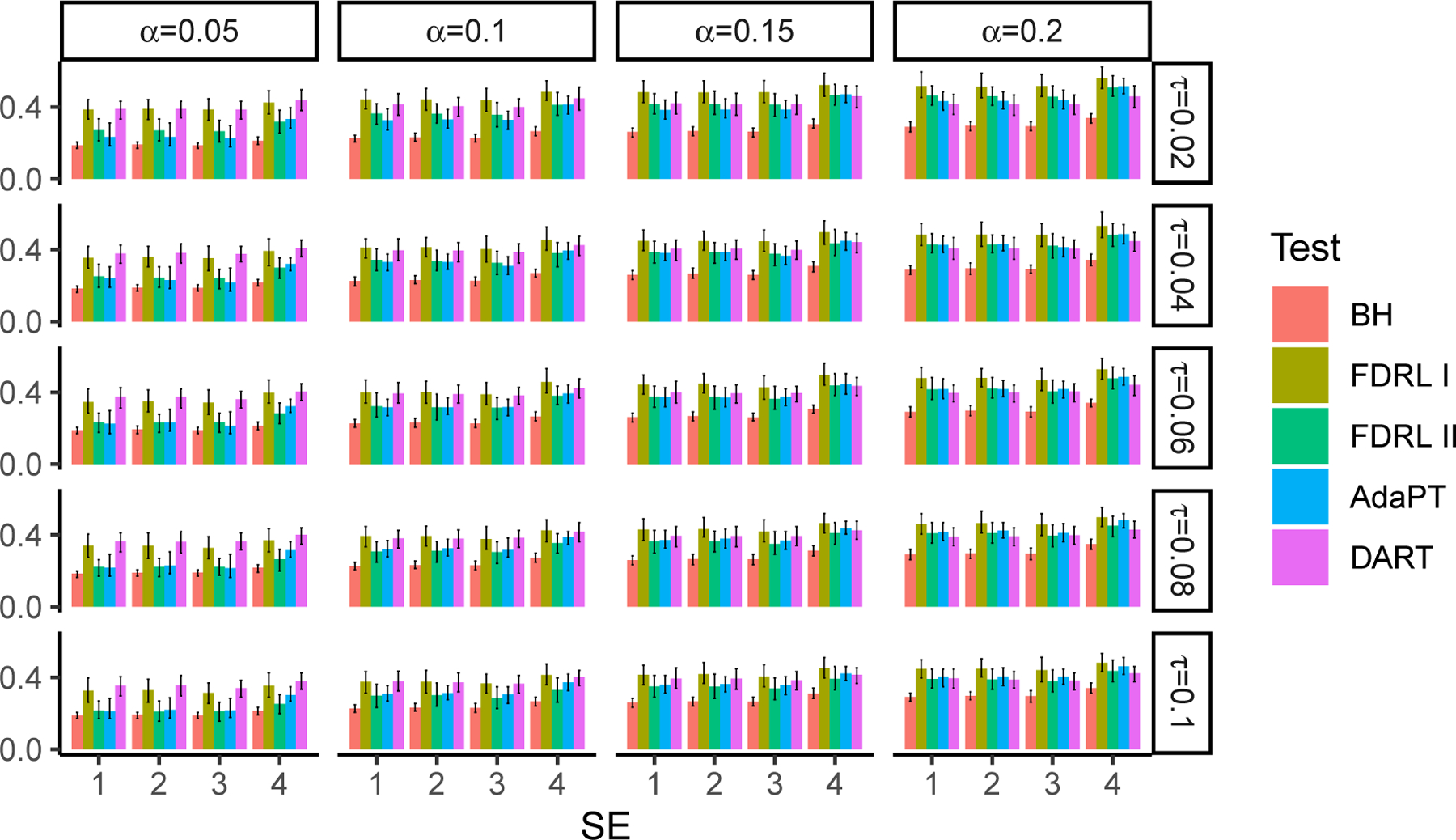
Sensitivity of various testing methods under SE1-SE4 when proportion τ of the alternative hypothesis were switched with the null. The main bars mark the average values across 200 repetitions; the error bars mark their 25% and 75% quantiles. Every column shows the sensitivity under a nominal FDR level α.

**Figure 6:**
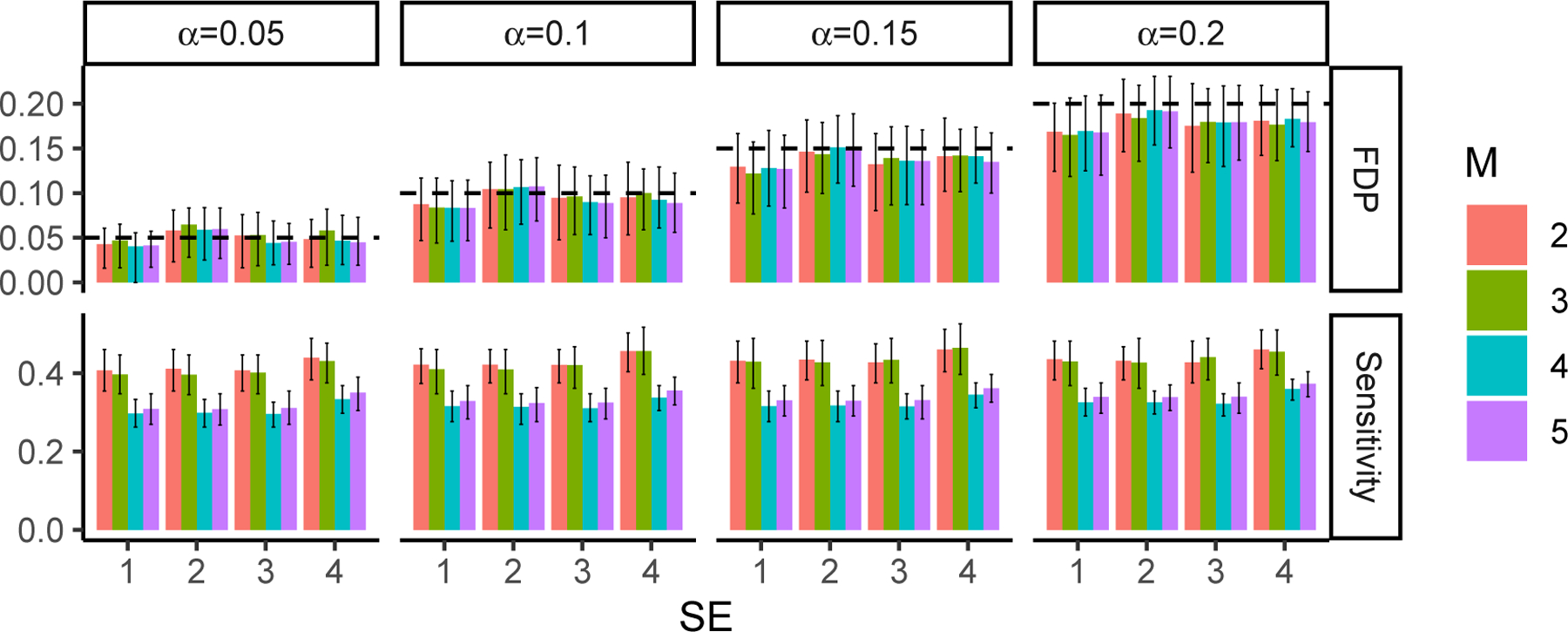
Performance of DART across different M. The main bars mark the average values across 200 repetitions; the error bars mark their 25% and 75% quantiles. Every column shows the performance under a nominal FDR level α. The first row represents the average FDP, and the dashed horizontal lines marks the desired FDR level. The second row represents the average sensitivity.

## Appendix Appendix A. More Definitions

In Section 4, we introduced a few terms, including moderate SNR nodes, strong SNR hypotheses, and weak SNR hypotheses. We provide their mathematical definitions here.

### Appendix A. 1 Moderate SNR Nodes

For any node A, we define its descendant set as

$$𝒟(A)=\left\{D:\exists \ell ,\;\text{such}\;\text{that}D\in {𝒜}^{\left(\ell \right)}\;\text{and}\;D⫋A\right\}\text{.}$$

The descendant set contains all the nodes from previous layers that a subset of A.

**Definition 4 (Moderate SNR node)** *A node A is called a moderate SNR node if*

(5)
$$\text{P}\left\{T_{A}<\alpha_{m},\forall D\in 𝒟(A),T_{D}\geq \overset{‾}{\Phi }\left(m^{r_{1}-1}\sqrt{\text{log}\;m}\right)\right\}\geq C_{1}>0,$$

To provide more intuitions on the moderate SNR nodes, we provide a sufficient condition for a node being a moderate SNR node when the test statistics are Gaussian-distributed.

**Example 1** *A sequence of independent Gaussian-distributed test statistics* $Z_{i}\sim \text{N}\left(\tau_{i},\;1\right)$ *for* i∈[m]. *To test the two-sided hypothesis*

$$H_{i}:\tau_{i}=0\;\text{versus}\;\tau_{i}\neq 0,$$

we derive the P-value $T_{i}=2\overset{‾}{\Phi }\left(\left|Z_{i}\right|\right)$. In example 1, if a node A satisfies

(6)
$$\forall i\in A,\;\left|\tau_{i}\right|\in \left(\frac{\gamma_{m}}{\sqrt{\left|A\right|}},\frac{\beta_{m}}{\sqrt{\left|A\right|-1}}\right)\;\text{with}\;\beta_{m}=\sqrt{2\left(1-r_{1}\right)\log\;m-2\;\text{log}\;\text{log}\;m}\;\text{and}\;\gamma_{m}=\sqrt{2\;\text{log}\;m+\;\text{log}\;\text{log}\;\text{log}m},$$

then A has moderate SNR. We request each $\tau_{i}$ falls in the range involving $\beta_{m}$ and $\gamma_{m}$; both slowly increase with m. In practice, the test statistics are calculated based on the observed samples. We usually consider the sample size n increases with the number of hypotheses m. As sample size n increases, the alternative SNR $\;\tau_{i}$ will also increase, often at the rate of $\sqrt{n}$. Thus, we usually expect to have some alternatives whose SNR falls into the range.

In (6), each $\tau_{i}$ needs to fall in the range. More generally, the purpose to define moderate SNR nodes is to define a set of alternative nodes (a) remaining in tree till they become candidate nodes, and (b) having large enough signals to be discovered when they become candidate nodes. We only need O(logm) such nodes to make sure the multiple testing procedure is stable and asymptotic valid.

### Appendix A. 2 Strong SNR Hypotheses

The definition of strong SNR hypotheses is linked with the definition of strong SNR node. On layer 1, we define strong SNR hypotheses. On layer ℓ with ℓ≥2, recursively, we exclude the strong SNR hypotheses defined on previous layers from each node, and then evaluate if this node is a strong SNR node; if so, all the hypotheses in this node are counted as strong SNR hypotheses. This process ends till reaching the top of the tree. Denote the strong SNR node set on layer ℓ by ${𝒢}_{\text{st}}^{\left(\ell \right)}$ and the strong SNR hypothesis set on layer ℓ by $\Omega_{\text{st}}^{\left(\ell \right)}$. To initiate, let $\Omega_{\text{st}}^{\left(0\right)}=\emptyset$.

**Definition 5 (Strong SNR hypotheses)** *On layer* ℓ, *after excluding the strong SNR hypotheses from the previous layers, define*

$${\tilde{𝒜}}^{\left(\ell \right)}=\left\{A\setminus \cup_{\ell^{\prime }=1}^{\ell -1}\Omega_{st}^{\left(\ell^{\prime }\right)}:A\in {𝒜}^{\left(\ell \right)}\right\}.$$

For any node $\tilde{A}\in {\tilde{𝒜}}^{\left(\ell \right)}$, if for all $i\in \tilde{A}$,

(7)
$$\text{P}\left\{T_{i}\in \kappa (|\tilde{A}|)\right\}>1-o\left(m^{-r_{1}}\right)\;\text{with}\;\kappa (|\tilde{A}|)=\left[m^{-\frac{1-r_{1}}{|\tilde{A}|-1}},{\left\{m(\text{log}\;\;m\;\text{log}\;\text{log}\;m{)}^{1/2}\right\}}^{-1/|\tilde{A}|}\right],$$

*Then* $\tilde{A}$ *is called a strong SNR node. The strong SNR hypothesis set on layer* ℓ *is*

$$\Omega_{st}^{\left(\ell \right)}=\left\{i\in \tilde{A}:\tilde{A}\;\text{is}\;\text{a}\;\text{strong}\;\text{SNR}\;\text{node}\;\text{in}\;{\tilde{𝒜}}^{\left(\ell \right)}\right\}.$$

The overall strong SNR hypothesis set is defined as $\Omega_{st}=\cup_{\ell =1}^{L-1}\Omega_{st}^{\left(\ell \right)}$.

With a high probability converging to 1, no hypothesis in $\Omega_{\text{st}}^{\left(\ell \right)}$ will be rejected before layer ℓ, but all of them will be rejected on layer ℓ.

Note that on layer ℓ≥2, it is possible that ${\left\{m(\text{log}\;m\text{log}\;\text{log}\;m{)}^{1/2}\right\}}^{-1/|\tilde{A}|}<m^{-\frac{1-r_{1}}{|𝒜|-1}}$, which leads $\kappa (|\tilde{A}|)=\emptyset$. In that case, strong SNR nodes do not exist. Our method does not require the existence of the strong SNR nodes.

Under Example 1, (7) is satisfied when the SNR

(8)
$$\left|\tau_{j}\right|\in \left(\frac{\gamma_{m}}{\sqrt{\left|\tilde{A}\right|}}+\lambda_{m},\frac{\beta_{m}}{\sqrt{\left|\tilde{A}\right|-1}}-\lambda_{m}\right)\;\text{with}\;\beta_{m},\gamma_{m}\;\text{defined}\;\text{in}\;(6),\lambda_{m}=\sqrt{2r_{1}\text{log}\;\;m}.$$

### Appendix A. 3 Weak SNR Hypotheses

Weak SNR hypotheses are those with weak SNRs so that they are very unlikely to be rejected if aggregated with other null hypotheses.

**Definition 6 (Weak SNR hypothesis)** *For any alternative hypothesis* $i\in \Omega_{1}$, *if*

(9)
$$\text{P}\left(T_{i}\in \iota \right)=o\left(m^{-r_{1}}\right)\;\text{with}\;\iota =\left(0,m^{\frac{r_{1}-1}{M^{L-1}}}\right),$$

this hypothesis is called a weak SNR hypothesis.

Under Example 1, (9) is satisfied if $\left|\tau_{i}\right|\in \left(0,\beta_{m}/\sqrt{M^{L-1}}\right)$.

## Appendix Appendix B. Proofs of Main Theorems

We introduce some notations before we provide the proofs. On layer ℓ, for a working node $S\in {ℬ}^{\left(\ell \right)}$, let $𝒰(S)=\left\{S^{\prime }\subset S:S^{\prime }\in \cup_{\ell^{\prime }=1}^{\ell -1}{ℬ}^{\left(\ell^{\prime }\right)}\right\}$ be the collection of sets in the testing path of S. In addition, let ${𝒰}^{c}(S)=\left\{S^{\prime \prime }\in \cup_{\ell^{\prime }=1}^{\ell -1}{ℬ}^{\left(\ell^{\prime }\right)}:S^{\prime \prime }\cap S=\emptyset ,S^{\prime \prime }\cup S\subset A\right.$, for some $\left.A\in {𝒜}^{\left(\ell \right)}\right\}$ be the collection of sets that was planning to combined with S on layer ℓ of the static aggregation tree but rejected on previous layers. When $S\in {ℬ}^{\left(1\right)}$, we set $𝒰(S)={𝒰}^{c}(S)=\emptyset$. We define $G_{S}(c)$ as the complementary CDF conditional on previous testing results. When ℓ=1, we have S={i}⊂{1,…,m}, and $G_{S}(c)=P\left(Z_{i}\geq c\right)$ with $Z_{1},\ldots ,Z_{m}\overset{iid}{\sim }N(0,1)$. When ℓ>1, the oracle rejection path for set $S\in {ℬ}^{\left(\ell \right)}$ is recursively defined as

$${𝒬}_{z}^{\left(1:\ell -1\right)}=\left\{z:\forall S^{\prime }\in 𝒰(S),G_{S^{\prime }}\left(Z_{S^{\prime }}\right)\geq {\overset{ˆ}{t}}^{\left(\ell_{S^{\prime }}\right)}(\alpha ),\forall S^{\prime \prime }\in {𝒰}^{c}(S),G_{S^{\prime \prime }}\left(Z_{S^{\prime \prime }}\right)\leq {\overset{ˆ}{t}}^{\left(\ell_{S^{\prime \prime }}\right)}(\alpha )\right\},$$

where

$$G_{S}(c)=\text{P}\left(Z_{S}\geq c|{𝒬}_{z}^{\left(1:\ell -1\right)}\right)$$

and $Z_{S}=\sum_{i\in S}\;Z_{i}/\sqrt{\left|S\right|}$, and $\ell_{S^{\prime }},\ell_{S^{\prime \prime }}\in \{1,\ldots ,\ell -1\}$ is the value s.t. $S^{\prime }\in {ℬ}^{\left(\ell_{S^{\prime }}\right)}$ and $S^{\prime \prime }\in {ℬ}^{\left(\ell_{S^{\prime \prime }}\right)}$, respectively.

Given $Z_{1},\ldots ,Z_{m}$ are mutually independent, we have

$$G_{S}(c)=\text{P}\left(Z_{S}\geq c|\forall S^{\prime }\in 𝒰(S),G_{S^{\prime }}\left(Z_{S^{\prime }}\right)\geq {\overset{ˆ}{t}}^{\left(\ell_{S^{\prime }}\right)}(\alpha )\right)$$

Given the definition of $G_{S}(c)$, we define the rejection path as

(10)
$${𝒬}^{\left(1:\ell -1\right)}=\left\{x:\forall S^{\prime }\in 𝒰(S),G_{S^{\prime }}\left(X_{S^{\prime }}\right)\geq {\overset{ˆ}{t}}^{\left(\ell_{S^{\prime }}\right)}(\alpha ),\forall S^{\prime \prime }\in {𝒰}^{c}(S),G_{S^{\prime \prime }}\left(X_{S^{\prime \prime }}\right)\leq {\overset{ˆ}{t}}^{\left(\ell_{S^{\prime \prime }}\right)}(\alpha )\right\}$$

In addition, for two sequence of real numbers $a_{m}$ and $b_{m}$, we write $a_{m}=o\left(b_{m}\right)$ when $a_{m}/b_{m}\to 0$, and $a_{m}=O\left(b_{m}\right)$ when ${\text{lim}}_{m\to \infty }\;\left|a_{m}/b_{m}\right|\leq C$ for some constant C. To prove the asymptotic properties of DART, we need the following lemmas. The proofs of lemmas are shown in supplementary materials.

**Lemma 7** *Let* ${𝒫}_{i}=\left\{p\in [0,\;1]:\text{P}\left({\tilde{T}}_{i}<p\right)\geq \epsilon (m)\right\}$ *and* ${𝒫}_{i}^{\prime }=\left\{p\in [0,\;1]:\text{P}\left({\tilde{T}}_{i}<p\right)\geq \right.\left.\;\epsilon (m)\epsilon^{\prime }(m)\right\}$, *with* $\epsilon (m),\epsilon^{\prime }(m)\to 0$. *For any set of independent random variable ${\overset{ˆ}{T}}_{i}\in [0,\;1]$*, *and a collection* $ℳ=\left\{S\subset \{1,\ldots ,m\}:|S|<c_{0}\right\}$ *with some constant* $c_{0}$,

1. *If* ${\text{max}}_{i\in ℳ}\;{\text{sup}}_{p\in {𝒫}_{i}^{\prime }}\;\left|\text{P}\left({\overset{ˆ}{T}}_{i}<p\right)/\text{P}\left({\tilde{T}}_{i}<p\right)-1\right|\to 0$, *then*,
  $$\underset{S_{0}\in ℳ}{\text{sup}}\;\underset{p\geq \epsilon (m)}{\text{sup}}\;\left|\frac{\text{P}\left(\sum_{i\in S_{0}}\;\;{\overset{ˆ}{X}}_{i}>c_{S_{0}}(p)\right)}{\text{P}\left(\sum_{i\in S_{0}}\;\;{\tilde{X}}_{i}>c_{S_{0}}(p)\right)}-1\right|\to 0,$$
2. *If* ${\text{lim}}_{m\to \infty }\;{\text{max}}_{i\in ℳ}\;{\text{sup}}_{p\in {𝒫}_{i}^{\prime }}\;\left(P\left({\overset{ˆ}{T}}_{i}<p\right)/\text{P}\left({\tilde{T}}_{i}<p\right)-1\right)\leq 0$, *then*,
  $$\underset{m\to \infty }{\text{lim}}\;\underset{S_{0}\in ℳ}{\text{sup}}\;\underset{p\geq \epsilon (m)}{\text{sup}}\;\left(\frac{\text{P}\left(\sum_{i\in S_{0}}\;\;{\overset{ˆ}{X}}_{i}>c_{S_{0}}(p)\right)}{\text{P}\left(\sum_{i\in S_{0}}\;\;{\tilde{X}}_{i}>c_{S_{0}}(p)\right)}-1\right)\leq 0$$

*Here*, ${\overset{ˆ}{X}}_{i}={\overset{‾}{\Phi }}^{-1}\left({\overset{ˆ}{T}}_{i}\right),{\tilde{X}}_{i}={\overset{‾}{\Phi }}^{-1}\left({\tilde{T}}_{i}\right)$ *and* $c_{S_{0}}(p)$ *is the value s.t*. $\text{P}\left[\sum_{i\in S_{0}}\;{\tilde{X}}_{i}>c_{S_{0}}(p)\right]=p$.

**Lemma 8** *Let* ${\tilde{\Omega }}_{0}=\left\{i:{\tilde{T}}_{i}\right.$ *follows* $\left.\text{Unif}\;(0,\;1)\right\},{ℬ}_{0a}^{\left(\ell \right)}≔\left\{S\in {ℬ}_{0}^{\left(\ell \right)}:\exists A\in {𝒜}^{\left(L\right)}\setminus {𝒜}^{\prime }\right.$, *s.t.S ⊂A*}, *and* ${ℬ}_{0b}^{\left(\ell \right)}≔\left\{S\in {ℬ}_{0a}^{\left(\ell \right)}:S\in {\tilde{\Omega }}_{0}\right\}$, *we have*:

1. $\underset{S\in {ℬ}_{0a}^{\left(\ell \right)}}{\text{max}}\;\underset{c\in \left[0,\gamma_{m}\right]}{\text{sup}}\;\left|\frac{G_{S}(c)}{\overset{‾}{\Phi }(c)}-1\right|\to 0$
2. $\underset{S\in {ℬ}_{0a}^{\left(\ell \right)}}{\text{max}}\;\;\underset{c\in \left[0,{\overset{‾}{\Phi }}^{-1}(1/m)\right]}{\text{sup}}\left|\frac{\text{P}\left(X_{S}>c|{𝒬}^{\left(1:\ell -1\right)}\right)}{\text{P}\left(X_{S}>c\right)}-1\right|\to 0$

**Lemma 9** *Define*

(11)
$${𝒳}^{\left(\ell \right)}\;=\left\{x:\sum_{S\in {ℬ}_{0}^{\left(\ell \right)}}\;\;|S|I\left(T_{S}<{\overset{ˆ}{t}}^{\left(\ell \right)}\right)-\sum_{S\in {ℬ}_{0}^{\left(\ell \right)}}\;\;|S|{\overset{ˆ}{t}}^{\left(\ell \right)}\leq \left\{\sum_{S\in {ℬ}_{0}^{\left(\ell \right)}}\;\;|S|{\overset{ˆ}{t}}^{\left(\ell \right)}\right\}\epsilon \right\}$$

$${𝒳}^{\prime (\ell )}\;=\left\{x:\left|\frac{\sum_{S\in {ℬ}_{0}^{\left(\ell \right)}}\;\;|S|I\left(T_{S}<{\overset{ˆ}{t}}^{\left(\ell \right)}\right)}{\sum_{S\in {ℬ}_{0}^{\left(\ell \right)}}\;\;|S|{\overset{ˆ}{t}}^{\left(\ell \right)}}-1\right|\geq \epsilon \right\}$$

*Then*, ∀ℓ=1,…,L, when the FDR control holds on layer 1,…,ℓ-1,

1. *For all* ϵ∈(0,α), *if* $\text{P}\left(m{\overset{ˆ}{t}}^{\left(\ell \right)}\geq Cc_{md}\right)\to 1$, *then* $\text{P}\left({𝒳}^{\left(\ell \right)}\right)=1-o(1)$. *Together with* ${\text{lim}}_{m\to \infty }\;\left|{\tilde{\Omega }}_{0}\right|/m=1$, *we have* $P\left({𝒳}^{\prime }(\ell )=1-o(1)\right.$.
2. *On* $\cap \cap_{h=1}^{\ell }{𝒳}^{\left(h\right)}$, *there exist a constant* C *s.t*. ${\overset{ˆ}{t}}^{\left(\ell \right)}\leq Cm^{r_{1}-1}$.
3. *Let* ${\overset{ˆ}{c}}_{S}$ *be the rejection threshold for the test node* $S\in {ℬ}^{\left(\ell \right)}$, *s.t*. ${\overset{‾}{G}}_{S}\left({\overset{ˆ}{c}}_{S}\right)={\overset{ˆ}{t}}^{\left(\ell \right)}$. *Then on* $\cap_{h=1}^{\ell }{𝒳}^{\left(h\right)}$,
  $${\overset{ˆ}{c}}_{S}>\beta_{m},\forall S\in {ℬ}^{\left(\ell \right)},$$
  *and on* $\cap_{h=1}^{\ell -1}{𝒳}^{\left(h\right)}$,
  $${\overset{ˆ}{c}}_{S}<\gamma_{m},\forall S\in {ℬ}^{\left(\ell \right)}.$$

Lemma 10

(12)
$$\frac{\sum_{S\in {ℬ}_{0}^{\left(\ell \right)}}\;\;|S|{\overset{ˆ}{t}}^{\left(\ell \right)}}{\sum_{S\in {ℬ}^{\left(\ell \right)}}\;\;|S|I\left(T_{S}<{\overset{ˆ}{t}}^{\left(\ell \right)}\right)}=\alpha (1+o(1))$$

**Proof [Proof of Theorem 3]** Let ${𝒱}^{\left(\ell \right)}=\left\{S\in {ℬ}_{0}^{\left(\ell \right)}:S\subset {ℛ}^{\left(\ell \right)}\right\}$ and ${𝒲}^{\left(\ell \right)}=\left\{S\in {ℬ}_{1}^{\left(\ell \right)}\right.\;:\;\left.S\subset {ℛ}^{\left(\ell \right)}\right\}$ be the false rejection node set and the rejection node set on layer ℓ, respectively. Define

$${𝒳}_{1}=\left\{S\in \cup_{\ell =2}^{L}{𝒲}^{\left(\ell \right)}:S\cap \Omega_{\text{st}}\neq \emptyset \;\text{and}\;S\cap \Omega_{0}\neq \emptyset \right\}\;{𝒳}_{2}=\left\{S\in \cup_{\ell =2}^{L}{𝒲}^{\left(\ell \right)}:S\cap \Omega_{\text{wk}}\neq \emptyset ,\;S\setminus \left(\Omega_{0}\cup \Omega_{\text{wk}}\right)=\emptyset \;\text{and}\;S\cap \Omega_{0}\neq \emptyset \right\}\;{𝒳}_{3}=\left\{S\in \cup_{\ell =2}^{L}{𝒲}^{\left(\ell \right)}:S\cap \Omega_{1}\setminus \left(\Omega_{\text{wk}}\cup \Omega_{\text{st}}\right)\neq \emptyset \;\text{and}\;S\cap \Omega_{0}\neq \emptyset \right\}$$

Then,

$$\text{P}\left({𝒳}_{1}\neq \emptyset \right)\leq \text{P}\left({𝒳}_{1}\neq \emptyset |\cap_{\ell =1}^{L}{𝒳}^{\left(\ell \right)}\right)\text{P}\left(\cap_{\ell =1}^{L}{𝒳}^{\left(\ell \right)}\right)+\text{P}\left({\left(\cap_{\ell =1}^{L}{𝒳}^{\left(\ell \right)}\right)}^{c}\right)\leq Cm^{r_{1}}o\left(m^{-r_{1}}\right)+o\left(1\right)\to 0\;\text{P}\left({𝒳}_{2}\neq \emptyset \right)\leq \text{P}\left({𝒳}_{2}\neq \emptyset |\cap_{\ell =1}^{L}{𝒳}^{\left(\ell \right)}\right)\text{P}\left(\cap_{\ell =1}^{L}{𝒳}^{\left(\ell \right)}\right)+\text{P}\left({\left(\cap_{\ell =1}^{L}{𝒳}^{\left(\ell \right)}\right)}^{c}\right)\;\overset{\left(a\right)}{\leq }Cm^{r_{1}}\text{P}\left[X_{S}\geq \beta_{m}|S\in \Omega_{\text{wk}}\cup \Omega_{0}\right]+o\left(1\right)\;\leq Cm^{r_{1}}o\left(m^{-r_{1}}\right)+o\left(1\right)\to 0$$

Here, the inequality (a) is based on Lemma 9 (1) and (3). By condition 4, $\left|{𝒳}_{3}\right|=o\left(c_{\text{md}}\right)$, accordingly,

$$\text{P}(FDP>\alpha +\epsilon )\;\leq \text{P}\left({𝒳}_{1}\cup {𝒳}_{2}\neq \emptyset \right)+\text{P}\left(\frac{\sum_{\ell =1}^{L}\sum_{S\in {𝒱}^{\left(\ell \right)}}\left|S\right|}{\sum_{\ell =1}^{L}\sum_{S\in {ℛ}_{\text{node}}^{\left(\ell \right)}}\left|S\right|}>\alpha +\epsilon ,{𝒳}_{1}\cup {𝒳}_{2}=\emptyset \right)\;\leq o\left(1\right)+\overset{L}{\underset{\ell =1}{\sum }}\text{P}\left(\frac{\sum_{S\in {𝒱}^{\left(\ell \right)}\setminus {𝒳}_{3}}\left|S\right|}{\sum_{S\in {ℛ}_{\text{node}}^{\left(\ell \right)}}\left|S\right|}>\alpha +\epsilon +o\left(1\right)\right)\to 0$$

So statement (a) is proved. The statement (b) can be proved in the similar way. ∎

## Appendix Appendix C. Additional Details on Simulation and Real Data Analysis

All the experiments were conducted on 2.10 GHz Intel Xeon Gold 6252 processors with 16 Gb memory at the Duke Compute Cluster. We requested 80 cores when running the simulation experiments to save time. Experiment code can be found in https://githubcom/jichunxie/DART_manu_support.git. We also built an R package, which can be found in https://github.com/jichunxie/DART.git.

### Appendix C. 1 Details on Numerical Experiments

We generated four simulation settings, each with n=300 observations on m=1000 features (hypotheses). All four simulation settings were based on a set of parameters $\eta_{i},i\in [m]$ related to alternative hypothesis signal levels. We defined two driver features 7 and 156; the features close to them were likely alternative. We also added 10 stand-alone features. Define the stand-alone feature set $\Omega_{2}=\{100,\;200,\ldots ,\;1000\}$.

$$\eta_{i}^{\prime }=\left\{\left[3.4\phi_{1}\left(d_{156,i}\right)-0.8\right]\vee 0\right\}+3\left\{\phi_{2}\left(d_{7,i}\right)\right\}+10*I\left(i\in \Omega_{2}\right),\;\eta_{i}=\eta_{i}^{\prime }I\left(\eta_{i}^{\prime }>0.15\right)$$

Here, $\phi_{1}$ and $\phi_{2}$ are the probability density functions of N(0,0.8) and N(0,0.05), respectively. Once feature locations and signals $\eta_{i}\text{s}$ were generated, they were fixed across all settings and all repetitions. We visualized the feature locations and their $\eta_{i}$ in Figure 4.

Below is the list of the four settings.

SE1: For node i∈{1,…,m}, we generated the P-values $T_{i}=2\overset{‾}{\Phi }\left(\left|{\overset{˘}{Z}}_{i}\right|\right)$ with ${\overset{˘}{Z}}_{1},\ldots ,{\overset{˘}{Z}}_{m}$ independently from $\text{N}\left(\sqrt{n}\theta_{i},1\right)$, with $\theta_{i}=\frac{1}{5}\eta_{i}$.
SE2: For node i∈{1,…,m}, we generated the P-values $T_{i}=2\overset{‾}{\Phi }\left(\left|{\overset{˘}{Z}}_{i}\right|\right)$ with ${\overset{˘}{Z}}_{1},\ldots ,{\overset{˘}{Z}}_{m}$ independently from the mixed Gaussian and T distribution $0.04t_{5}\left(\sqrt{n}\theta_{i}\right)+0.96\;\text{N}\left(\sqrt{n}\theta_{i},1\right)$. Here, ${\text{t}}_{5}\;\left(\sqrt{n}\theta_{i}\right)$ stands for the student t distribution with the degree of freedom 5 and none centrality parameter $\sqrt{n}\theta_{i}$, with $\theta_{i}=\frac{1}{5}\eta_{i}$.
SE3: Consider the linear mode $Y_{i}=\vartheta_{0,i}+\theta_{i}W_{1}+\vartheta_{2,i}W_{2}+\epsilon_{i}$. Here, $W_{1}$ and $W_{2}$ were generated from Binom(0.5) and $\text{Unif}\;(0.1,\;0.5);\epsilon_{i}$ was from N(0,1). We set $\vartheta_{0,i}=\vartheta_{2,i}=0.1$ and $\theta_{i}=\frac{2}{5}\eta_{i}$. The P-values $T_{i}$ were generated from the Wald test of testing whether $\theta_{i}$ is zero.
SE4: We generated the data $𝒟=\left\{\left(W_{1,j},W_{2,j},\Delta_{ij},E_{ij}\right):i\in [m],j\in [n]\right\}$. Here, the covariates $W_{1,j}$ and $W_{2,j}$ were sampled from Binom(0.5) and Unif(0.1,0.5), respectively. $E_{ij}\in [0,+\infty )$ is the survival time and $\Delta_{ij}\in \{0,\;1\}$ is the event indicator. The true event time was generated from the exponential distribution with the rate $\text{exp}\;\left\{\theta_{i}W_{1,j}+\vartheta_{i}W_{2,j}\right\}$, where $\vartheta_{i}=0.1$ and $\theta_{i}=\frac{1}{2}\eta_{i}$. The centering time $C_{ij}$ was generated from Unif(0,5). The observed event time was set as $E_{ij}=\text{min}\left\{{\tilde{E}}_{ij},C_{ij}\right\}$. We used the Cox regression model to regress the covariates $W_{1,j}$ and $W_{2,j}$ on the event $\left(\Delta_{ij},E_{ij}\right)$ Cox (1972). The P-values $T_{i}$ were generated from the Wald test of testing whether $\theta_{i}$ is zero.

To check the robustness of the algorithms when the distance cannot fully reflect the co-status patterns, we switched some hypotheses’ null/alternative statuses. In particular, we randomly changed proportion τ of alternative hypotheses to be null and proportional τ of the null hypotheses to be alternative. The switching rate τ reflects the violation level of the co-status patterns. Here is the detailed switching process.

1. Denote the original null and alternative sets by $\Omega_{0}^{\prime }=\left\{i:\theta_{i}=0\right\}$ and $\Omega_{1}^{\prime }=\left\{i:\theta_{i}>0\right\}$.
2. We randomly selected the elements from the original null and alternative sets to form
  - the alternative-to-null set $\Omega_{1,0}^{\prime }\subset \Omega_{1}^{\prime }$, where $\left|\Omega_{1,0}^{\prime }\right|=\left\lfloor \tau \left|\Omega_{1}^{\prime }\right|\right\rfloor$; and
  - the null-to-alternative set $\Omega_{0,1}^{\prime }\subset \Omega_{0}^{\prime }$, where $\left|\Omega_{0,1}^{\prime }\right|=\left|\Omega_{1,0}^{\prime }\right|$
3. Re-assign the signal parameters.
  - For $i\in \Omega_{0,1}^{\prime }$, re-assign $\theta_{i}=\theta_{j}$ for a random-chosen $j\in \Omega_{1,0}^{\prime }$.
  - For $i\in \Omega_{1,0}^{\prime }$, set $\theta_{i}=0$.
4. Define the new null and alternative sets $\Omega_{0}=\Omega_{0}^{\prime }\cup \Omega_{1,0}^{\prime }\setminus \Omega_{0,1}^{\prime }$ and $\Omega_{1}=\Omega_{1}^{\prime }\cup \Omega_{0,1}^{\prime }\setminus \Omega_{1,0}^{\prime }$.

The average FDP and sensitivity across 200 repetitions were summarized in Figure 2B and Figure 5.

We applied DART, BH (Benjamini and Hochberg, 1995), two ${\text{FDR}}_{L}$ procedures (Zhang et al., 2011), and AdaPT (Lei and Fithian, 2018) to the above numerical settings. BH does not have tuning parameters. For DART, based on the tuning parameter selection criterion in Section 3.4, we set M=3 and constructed a 3-layer aggregation tree, with distance thresholds $g^{\left(2\right)}=0.88$ and $g^{\left(3\right)}=1.52$. For the two ${\text{FDR}}_{L}$ procedures, Zhang et al. (2011) recommended setting k as an odd number greater than three and used k=5 in the simulation experiments. Thus, we set k=5 in our numerical experiments too. For AdaPT, we followed the instructions found at https://cran.r-project.org/web/packages/adaptMT/vignettes/adapt_demo.html to set up its tuning parameters. During simulation, we noticed that AdaPT sometimes failed to generate results after a long execution time. To ensure valid results from AdaPT, Figures 2 and 5 only summarize the repetition who successfully deliver a result. Table 1 lists the number of repetitions that failed under different scenarios among 200 repetitions.

**Table 1:** Number of repetition fails to deliver testing result within 8 minutes.

SE	τ=0	τ=0.02	τ=0.04	τ=0.06	τ=0.08	τ=0.1
1	33	41	34	45	46	45
2	34	37	29	42	39	45
3	23	30	36	42	32	51
4	12	19	15	27	25	36

### Appendix C. 2 Experiments to Decide the Optimal Maximum Node Size

We considered four settings of the maximum node sizes: M∈{2,3,4,5}. To design a good tuning parameter selection criterion, we evaluated DART’s performance with different M via numerical experiments described in Section 5. The maximum number of layers L and distance thresholds $g^{\left(\ell \right)}$ were set according to Section 3.4 and Algorithm 3. We listed the resulting tuning parameters in Table 2

Figure 6 shows that no matter what M we used, DART always controls the average FDR well under SE1, SE3, and SE4. For SE2, when the nominal FDR is 5%, DART has slight inflation because SE2 deliberately misspecified the null distribution. However, when the nominal FDR ≥ 10%, DART under SE2 still has the average FDP well controlled. This indicates that DART’s performance is robust in M. When M∈{2,3}, DART has higher sensitivity. Thus, in practice, we recommend using M=2 or M=3.

**Table 2:** Summary of the tuning parameters selected under different M

M	2	3	4	5
Maximum Layer L	4	3	2	2
Distance thresholds	$g^{\left(2\right)}=1.20$ $g^{\left(3\right)}=1.52$ $g^{\left(4\right)}=1.74$	$g^{\left(2\right)}=0.88$ $g^{\left(3\right)}=1.52$	$g^{\left(2\right)}=0.25$	$g^{\left(2\right)}=0.19$

### Appendix C. 3 Aggregation Tree Construction in Real-world Experiment

Because 9 ASVs are chosen as the reference ASVs, the distance matrix is calculated among the remaining 857 non-reference ASV using the R package Phangorn (Schliep, 2011) based on the JC69 model (Jukes et al., 1969). As the default model in Phangorn, the JC69 model is a classical Markov model of DNA sequence evolution and can be used to estimate the evolutionary distance between sequences. Two ASVs with similar sequences tend to be evolutionary close to each other, and more likely to perform similar biological functions. Therefore, we incorporate the distance matrix in identifying the important ASVs.

Based on the tuning parameter selection procedure described in Section 2.3, we construct an aggregation tree with M=3,L=3. The set of possible threshold G is set as $\{4,\;8,\;12,\;16\}/\sqrt{n\text{log}\;m\text{log}\;\text{log}\;m}$, with n=456 and m=857, and we choose $g^{\left(2\right)}=g^{\left(3\right)}=16/\sqrt{n\text{log}\;m\text{log}\;\text{log}\;m}=0.21$.

## References

[R1] Aitchison John. The statistical analysis of compositional data. Journal of the Royal Statistical Society: Series B, 44(2):139–160, 1982.

[R2] Alexander Aaron F, Vértes Petra E, Stidd Reva, Lalonde François, Clasen Liv, Rapoport Judith, Giedd Jay, Bullmore Edward T, and Gogtay Nitin. The anatomical distance of functional connections predicts brain network topology in health and schizophrenia. Cereb Cortex, 23(1):127–38, Jan 2013. doi: 10.1093/cercor/bhr388. 22275481 PMC3513955

[R3] Belomestny Denis and Spokoiny Vladimir. Spatial aggregation of local likelihood estimates with applications to classification. The Annals of Statistics, 35(5):2287–2311, 2007. ISSN 00905364. URL http://www.jstor.org/stable/25464582.

[R4] Benjamini Yoav and Hochberg Yosef. Controlling the false discovery rate: a practical and powerful approach to multiple testing. Journal of the Royal Statistical Society: Series B, 57(1):289–300, 1995.

[R5] Blondel Vincent D, Guillaume Jean-Loup, Lambiotte Renaud, and Lefebvre Etienne. Fast unfolding of communities in large networks. Journal of Statistical Mechanics: Theory and Experiment, 2008(10):P10008, oct 2008. doi: 10.1088/1742-5468/2008/10/p10008.

[R6] Cai T Tony and Liu Weidong. Large-scale multiple testing of correlations. Journal of the American Statistical Association, 111(513):229–240, 2016a. doi: 10.1080/01621459.2014.999157. 27284211 PMC4894362

[R7] Cai T Tony and Liu Weidong. Large-scale multiple testing of correlations. Journal of the American Statistical Association, 111(513):229–240, 2016b. 27284211 10.1080/01621459.2014.999157 PMC4894362

[R8] Cai T Tony, Sun Wenguang, and Xia Yin. Laws: A locally adaptive weighting and screening approach to spatial multiple testing. Journal of the American Statistical Association, pages 1–30, 2020.

[R9] Callahan Benjamin J, McMurdie Paul J, Rosen Michael J, Han Andrew W, Johnson Amy Jo A, and Holmes Susan P. Dada2: high-resolution sample inference from illumina amplicon data. Nature Methods, 13(7):581, 2016. 27214047 10.1038/nmeth.3869 PMC4927377

[R10] Chen Jun, Bittinger Kyle, Charlson Emily S, Hoffmann Christian, Lewis James, Wu Gary D, Collman Ronald G, Bushman Frederic D, and Li Hongzhe. Associating microbiome composition with environmental covariates using generalized unifrac distances. Bioinformatics, 28(16):2106–13, Aug 2012. doi: 10.1093/bioinformatics/bts342. 22711789 PMC3413390

[R11] Claesson Marcus J, Jeffery Ian B, Conde Susana, Power Susan E, O’connor Eibhlís M, Cusack Siobhán, Harris Hugh MB, Coakley Mairead, Lakshminarayanan Bhuvaneswari, O’Sullivan Orla, Gut microbiota composition correlates with diet and health in the elderly. Nature, 488(7410):178–184, 2012. 22797518 10.1038/nature11319

[R12] Cox David R. Regression models and life-tables. Journal of the Royal Statistical Society: Series B, 34(2):187–202, 1972.

[R13] Dmitrienko Alex and Tamhane Ajit C. General theory of mixture procedures for gatekeeping. Biometrical Journal, 55(3):402–19, May 2013. doi: 10.1002/bimj.201100258. 23423821

[R14] Dmitrienko Alex, Wiens Brian, Tamhane Ajit, and Wang Xin. Tree-structured gatekeeping tests in clinical trials with hierarchically ordered objectives. Statistics in Medicine, 26: 2465–78, 05 2007. doi: 10.1002/sim.2716. 17054103

[R15] Dmitrienko Alex, Tamhane Ajit C, Liu Lingyun, and Wiens Brian L. A note on tree gatekeeping procedures in clinical trials. Statistics in Medicine, 27(17):3446–51, Jul 2008. doi: 10.1002/sim.3307. 18484599

[R16] Dmitrienko Alex, Kordzakhia George, and Tamhane Ajit C. Multistage and mixture parallel gatekeeping procedures in clinical trials. Journal of Biopharmaceutical Statistics, 21(4): 726–47, Jul 2011. doi: 10.1080/10543406.2011.551333. 21516566

[R17] Eklund Anders, Andersson Mats, Josephson Camilla, Johannesson Magnus, and Knutsson Hans. Does parametric fmri analysis with spm yield valid results? an empirical study of 1484 rest datasets. Neuroimage, 61(3):565–78, Jul 2012. doi: 10.1016/j.neuroimage.2012.03.093. 22507229

[R18] Fisher Ronald. Statistical method for research workers. Oliver and Boyd, Edinburgh ;London, 1925.

[R19] Garcia Tanya P, Müller Samuel, Carroll Raymond J, and Walzem Rosemary L. Identification of important regressor groups, subgroups and individuals via regularization methods: application to gut microbiome data. Bioinformatics, 30(6):831–7, Mar 2014. doi: 10.1093/bioinformatics/btt608. 24162467 PMC3957069

[R20] Goeman Jelle J and Finos Livio. The inheritance procedure: multiple testing of tree-structured hypotheses. Statistical Applications in Genetics and Molecular Biology, 11(1): Article 11, Jan 2012. doi: 10.1515/1544-6115.1554. 22499687

[R21] Jenq Robert R, Ubeda Carles, Taur Ying, Menezes Clarissa C, Khanin Raya, Dudakov Jarrod A, Liu Chen, West Mallory L, Singer Natalie V, Equinda Michele J, Regulation of intestinal inflammation by microbiota following allogeneic bone marrow transplantation. Journal of Experimental Medicine, 209(5):903–911, 2012. 22547653 10.1084/jem.20112408 PMC3348096

[R22] Jin Xin and Han Jiawei. K-Means Clustering, pages 563–564. Springer US, Boston, MA, 2010. ISBN 978–0-387–30164-8. doi: 10.1007/978-0-387-30164-8_425.

[R23] Jukes Thomas H, Cantor Charles R, Evolution of protein molecules. Mammalian Protein Metabolism, 3:21–132, 1969.

[R24] Kristanto Daniel, Liu Mianxin, Liu Xinyang, Sommer Werner, and Zhou Changsong. Predicting reading ability from brain anatomy and function: From areas to connections. Neuroimage, 218:116966, 09 2020. doi: 10.1016/j.neuroimage.2020.116966. 32439534

[R25] Kurtz Zachary D, Müller Christian L, Miraldi Emily R, Littman Dan R, Blaser Martin J, and Bonneau Richard A. Sparse and compositionally robust inference of microbial ecological networks. PLoS Computational Biology, 11(5), 2015. 10.1371/journal.pcbi.1004226 PMC4423992 25950956

[R26] Lee Donghwan and Lee Youngjo. Extended likelihood approach to multiple testing with directional error control under a hidden markov random field model. Journal of Multivariate Analysis, 151:1 – 13, 2016. ISSN 0047–259X. doi: 10.1016/j.jmva.2016.07.001.

[R27] Lei Lihua and Fithian William. Adapt: an interactive procedure for multiple testing with side information. Journal of the Royal Statistical Society: Series B, 80(4):649–679, 2018.

[R28] Lei Lihua, Ramdas Aaditya, and Fithian William. A general interactive framework for false discovery rate control under structural constraints. Biometrika, 108(2):253–267, 07 2020. ISSN 0006–3444. doi: 10.1093/biomet/asaa064.

[R29] Li Xiaoqing, Lin Yu, Li Xue, Xu Xiaoxiao, Zhao Yanmin, Xu Lin, Gao Yang, Li Yixue, Tan Yamin, Qian Pengxu, Tyrosine supplement ameliorates murine agvhd by modulation of gut microbiome and metabolome. EBioMedicine, 61:103048, 2020a. 33039712 10.1016/j.ebiom.2020.103048 PMC7553238

[R30] Li Yimei, Gilmore John H, Shen Dinggang, Styner Martin, Lin Weili, and Zhu Hongtu. Multiscale adaptive generalized estimating equations for longitudinal neuroimaging data. Neuroimage, 72:91–105, May 2013. doi: 10.1016/j.neuroimage.2013.01.034. 23357075 PMC3621129

[R31] Li Yunxiao, Hu Yi-Juan, and Satten Glen A.. A bottom-up approach to testing hypotheses that have a branching tree dependence structure, with error rate control. Journal of the American Statistical Association, pages 1–18, sep 2020b. doi: 10.1080/01621459.2020.1799811. PMC9269868 35814292

[R32] Lin Dandan, Hu Bo, Li Pengfei, Zhao Ye, Xu Yang, and Wu Depei. Roles of the intestinal microbiota and microbial metabolites in acute gvhd. Experimental Hematology & Oncology, 10(1):1–19, 2021. 34706782 10.1186/s40164-021-00240-3 PMC8555140

[R33] Liu Weidong Gaussian graphical model estimation with false discovery rate control. The Annals of Statistics, 41(6):2948–2978, 2013.

[R34] Liu Yaowu and Xie Jun. Cauchy combination test: a powerful test with analytic p-value calculation under arbitrary dependency structures. Journal of the American Statistical Association, 115(529):393–402, 2020. doi: 10.1080/01621459.2018.1554485. 33012899 PMC7531765

[R35] Loper Jackson H, Lei Lihua, Fithian William, and Tansey Wesley. Smoothed nested testing on directed acyclic graphs. Biometrika, 109(2):457–471, 2022. 38694183 10.1093/biomet/asab041 PMC11061840

[R36] Martiny Jennifer B H, Jones Stuart E, Lennon Jay T, and Martiny Adam C. Microbiomes in light of traits: A phylogenetic perspective. Science, 350(6261):aac9323, Nov 2015. doi: 10.1126/science.aac9323. 26542581

[R37] Meijer Rosa J and Goeman Jelle J. A multiple testing method for hypotheses structured in a directed acyclic graph. Biometrical Journal, 57(1):123–143, 2015. 25394320 10.1002/bimj.201300253

[R38] Payen Mathilde, Nicolis Ioannis, Robin Marie, Michonneau David, Delannoye Johanne, Mayeur Camille, Kapel Nathalie, Berçot Béatrice, Butel Marie-José, Le Goff Jérôme, Functional and phylogenetic alterations in gut microbiome are linked to graft-versus-host disease severity. Blood Advances, 4(9):1824–1832, 2020. 32353108 10.1182/bloodadvances.2020001531 PMC7218439

[R39] Perinelli Alessio, Tabarelli Davide, Miniussi Carlo, and Ricci Leonardo. Dependence of connectivity on geometric distance in brain networks. Scientific Reports, 9(1):13412, 09 2019. doi: 10.1038/s41598-019-50106-2. 31527782 PMC6746748

[R40] Ramdas Aaditya, Chen Jianbo, Wainwright Martin J, and Jordan Michael I. A sequential algorithm for false discovery rate control on directed acyclic graphs. Biometrika, 106(1): 69–86, 2019a.

[R41] Ramdas Aaditya K., Barber Rina F., Wainwright Martin J., and Jordan Michael I.. A unified treatment of multiple testing with prior knowledge using the p-filter. The Annals of Statistics, 47(5), oct 2019b. doi: 10.1214/18-aos1765.

[R42] Ren Zhimei and Candès Emmanuel. Knockoffs with side information. 01 2020. URL https://arxiv.org/pdf/2001.07835.pdf.

[R43] Schliep KP. phangorn: phylogenetic analysis in r. Bioinformatics, 27(4):592–593, 2011. URL 10.1093/bioinformatics/btq706. 21169378 PMC3035803

[R44] Shono Yusuke, Docampo Melissa D, Peled Jonathan U, Perobelli Suelen M, Velardi Enrico, Tsai Jennifer J, Slingerland Ann E, Smith Odette M, Young Lauren F, Gupta Jyotsna, Increased gvhd-related mortality with broad-spectrum antibiotic use after allogeneic hematopoietic stem cell transplantation in human patients and mice. Science Translational Medicine, 8(339):339ra71–339ra71, 2016. 10.1126/scitranslmed.aaf2311 PMC4991773 27194729

[R45] Shu Hai, Nan Bin, and Koeppe Robert. Multiple testing for neuroimaging via hidden markov random field. Biometrics, 71(3):741–750, 2015. 26012881 10.1111/biom.12329 PMC4579542

[R46] Soriano J and Ma L. Probabilistic multi-resolution scanning for two-sample differences. Journal of The Royal Statistical Society Series B-statistical Methodology, 79:547–572, 2017.

[R47] Tansey Wesley, Wang Yixin, Blei David, and Rabadan Raul. Black box FDR. In International conference on machine learning, pages 4867–4876. PMLR, 2018.

[R48] Traag Vincent A., Waltman Ludo, and van Eck Nees Jan. From louvain to leiden: guaranteeing well-connected communities. Scientific Reports, 9, 2019. 10.1038/s41598-019-41695-z PMC6435756 30914743

[R49] Turek Matt. Explainable artifical intelligence (xai). https://www.darpa.mil/program/explainable-artificial-intelligence, 2021.

[R50] Xi Dong and Tamhane Ajit C. A general multistage procedure for k-out-of-n gatekeeping. Statistics in Medicine, 33(8):1321–35, Apr 2014. doi: 10.1002/sim.6028. 24307257

[R51] Xia Fei, Zhang Martin J, Zou James Y, and Tse David. Neuralfdr: Learning discovery thresholds from hypothesis features. Advances in neural information processing systems, 30,2017.

[R52] Xie Jichun and Li Ruosha. False discovery rate control for high dimensional networks of quantile associations conditioning on covariates. Journal of the Royal Statistical Society: Series B, 80(5):1015–1034, Nov 2018. doi: 10.1111/rssb.12288. PMC6497089 31057329

[R53] Yekutieli Daniel. Hierarchical false discovery rate-controlling methodology. Journal of the American Statistical Association, 103(481):309–316, 2008. ISSN 01621459. URL http://www.jstor.org/stable/27640041.

[R54] Zepeda-Mendoza Marie Lisandra and Resendis-Antonio Osbaldo. Hierarchical Agglomerative Clustering, pages 886–887. Springer New York, New York, NY, 2013. ISBN 978–1-4419–9863-7. doi: 10.1007/978-1-4419-9863-7_1371.

[R55] Zhang Chunming, Fan Jianqing, and Yu Tao. Multiple testing via ${\text{FDR}}_{L}$ for large scale imaging data. Annals of Statistics, 39(1):613, 2011. 21643445 10.1214/10-AOS848SUPP PMC3105786

