# Supplementary material for: DART: Distance Assisted Recursive Testing
Source: J Mach Learn Res. Author manuscript; Available in PMC 2024 Dec 12. (PMC11636646)
Supplement: 1 [file NIHMS1998821-supplement-1.pdf]

Supplementary Materials for "DART: Distance Assisted Recursive Testing"

Appendix S1. Proofs of Lemmas

Proof [Proof of Lemma 1] Since the proof of the theorem statement (b) is similar to the proof of the theorem statement (a), we will only focusing on the proof of statement (a).

The random variable $FDP^{(\ell)}$ can be decomposed to the product of two parts.

$$FDP^{(\ell)} = \frac{\sum_{S\in\mathcal{B}_0^{(\ell)}}|S|I\{T_S < \hat{t}^{(\ell)}\}}{\sum_{S\in\mathcal{B}_0^{(\ell)}}|S|\hat{t}^{(\ell)}} \times \frac{\sum_{S\in\mathcal{B}_0^{(\ell)}}|S|\hat{t}^{(\ell)}}{\max(\sum_{S\in\mathcal{B}^{(\ell)}}|S|I\{T_S < \hat{t}^{(\ell)}\}, 1)} \tag{S1}$$

Based on (S1), in order to prove $\lim_{m\to\infty}\mathrm{P}(FDP^{(\ell)} \le \alpha + \epsilon) = 1$ for all $\epsilon > 0$, we only need prove

$$\lim_{m\to\infty} \mathrm{P}\left\{\frac{\sum_{S\in\mathcal{B}_0^{(\ell)}}|S|I\{T_S < \hat{t}^{(\ell)}\}}{\sum_{S\in\mathcal{B}_0^{(\ell)}}|S|\hat{t}^{(\ell)}} - 1 < \epsilon\right\} \to 1 \tag{S2}$$

$$\lim_{m\to\infty} \mathrm{P}\left\{\left|\frac{\sum_{S\in\mathcal{B}_0^{(\ell)}}|S|\hat{t}^{(\ell)}}{\max(\sum_{S\in\mathcal{B}^{(\ell)}}|S|I\{T_S < \hat{t}^{(\ell)}\}, 1)} - \alpha\right| > \epsilon\right\} \to 0 \tag{S3}$$

(S3) is immediately followed by Lemma 10, and we will prove (S2) by induction. Below is a list of the proof sketch:

1. On layer 1, show $P(m\hat{t}^{(1)} \ge Cc_{\text{md}}) \to 1$. Then, by applying Lemma 9, we have
   - $P(\mathcal{X}^{(1)}) \to 1$, which is equivalent to (S2). Hence, we proved the FDR control on layer 1.
   - $P(\beta_m < \hat{c}_S < \gamma_m, \forall S \in \mathcal{B}^{(1)}) \to 1$, and $P(\hat{c}_S < \gamma_m, \forall S \in \mathcal{B}^{(2)}) \to 1$. Note that although this conclusion is not used to prove the FDR control on the current layer, but is necessary to guarantee the FDR control on higher layers.
2. On layer $\ell \ge 2$, assume the FDR control holds on previous layers and $P(\mathcal{X}^{(\ell')}) \to 1$ for all $\ell' = 1, \ldots, \ell - 1$. Then by Lemma 9, $P(\beta_m < \hat{c}_S < \gamma_m, \forall S \in \cup_{\ell'=1}^{\ell-1}\mathcal{B}^{(\ell')}) \to 1$, and $P(\hat{c}_S < \gamma_m, \forall S \in \mathcal{B}^{(\ell)}) \to 1$. Accordingly, we can get $P(m\hat{t}^{(1)} \ge Cc_{\text{md}}) \to 1$. Then, by applying the Lemma 9 again, we have
   - $P(\mathcal{X}^{(\ell)}) \to 1$, which is equivalent to (S2). Hence, we proved the FDR control on layer ℓ.
   - $P(\beta_m < \hat{c}_S < \gamma_m, \forall S \in \mathcal{B}^{(\ell)}) \to 1$, and $P(\hat{c}_S < \gamma_m, \forall S \in \mathcal{B}^{(\ell+1)}) \to 1$.

We start the proof on layer 1. **Layer 1:**

Take a subset $\mathcal{F}^{(1)} \subset \mathcal{A}_{\text{md}} \cap \mathcal{A}^{(1)}$, such that $|\mathcal{F}^{(1)}| = c_{\text{md}}$. For any $i \in \mathcal{F}^{(1)}$, we have $\mathrm{P}(X_i > \gamma_m) \ge C$. By Markov's inequality, we have:

$$\mathrm{P}\left(\Big|\sum_{i\in\mathcal{F}^{(1)}} I(X_i > \gamma_m) - \sum_{i\in\mathcal{F}^{(1)}} \mathrm{P}(X_i > \gamma_m)\Big| \ge c_{\text{md}}^{3/4}\right) \le C(c_{\text{md}})^{-1/2}$$

Thus,

$$\mathrm{P}\big[\sum_{1\le i\le m} I(T_i \le \hat{t}^{(1)}) \ge Cc_{\mathrm{md}} - c_{\mathrm{md}}^{3/4}\big] \ge 1 - o(1)$$

Therefore, by Lemma 10, exists constant $C^{(1)}$, s.t.

$$\mathrm{P}\left[m_0\hat{t}^{(1)} \ge C^{(1)}c_{\mathrm{md}}\right] \ge 1 - o(1) \tag{S4}$$

Together with Lemma 9 (1), we have $\mathrm{P}(\mathcal{X}^{(1)}) \to 1$ and accordingly, $\mathrm{P}(FDP^{(1)} < \alpha + \epsilon) \to 1$.

Layer ℓ:

Based on similar arguments on Layer 1, it suffices to show $\mathrm{P}(m_0\hat{t}^{(\ell)} > C^{(\ell)}c_{\mathrm{md}}) \to 1$ for some constant $C^{(\ell)}$.

Assume $\forall h = 1, \dots, \ell-1$, $\mathrm{P}(\mathcal{X}^{(h)}) \to 1$, then by Lemma 9, we have $\mathrm{P}(\beta_m < \hat{c}_S < \gamma_m, \forall S \in \mathcal{B}^{(h)}) \to 1$, and $\mathrm{P}(c_S < \gamma_m, \forall S \in \mathcal{B}^{(\ell)}) \to 1$.

Let $\mathcal{F}^{(\ell)} \subset \mathcal{A}_{\mathrm{md}} \cap \mathcal{A}^{(\ell)}$ with $|\mathcal{F}^{(\ell)}| = c_{\mathrm{md}}$. Define

$$\hat{\mathcal{F}}^{(\ell)} = \left\{A \in \mathcal{B}^{(\ell)} \cap \mathcal{F}^{(\ell)} : T_A < \alpha_m\right\}$$

By condition 2, $\forall A \in \mathcal{F}^{(\ell)}$,

$$\mathrm{P}(A \in \hat{\mathcal{F}}^{(\ell)}) \ge \mathrm{P}\left(T_A < \alpha_m, T_D \ge \bar{\Phi}(m^{r_1-1}\sqrt{\log m}), \forall D \in \mathcal{D}(A)\right) \ge C_1 \tag{S5}$$

Accordingly, define $\hat{\mathcal{X}}^{(\ell)} = \{|\hat{\mathcal{F}}^{(\ell)}| \ge c_{\mathrm{md}}/2\}$, then $\mathrm{P}(\hat{\mathcal{X}}^{(\ell)}) \ge 1 - o(1)$.

On $\hat{\mathcal{X}}^{(\ell)}$, we have

$$\sum_{S\in\mathcal{B}_1^{(\ell)}} I(T_S \le \hat{t}^{(\ell)}) \ge Cc_{\mathrm{md}}$$

Then based on Lemma 8, we can conclude that $\mathrm{P}(m_0\hat{t}^{(\ell)} \ge C^{(\ell)}c_{\mathrm{md}}) \ge 1 - o(1)$ for some constant $C^{(\ell)}$. ■

Proof [Proof of Lemma 7] (1) Define $\tilde{X}_i = \bar{\Phi}(\tilde{T}_i)$, For $k \in \{1, ..., c_0\}$, let $q_0 \ge \epsilon(m)$. Also define $b_{1,k}(q_0)$, c_1,...,c_k be the value s.t. $P(\sum_{j=1}^k \tilde{X}_j > b_{1,k}(q_0)) = q_0\left[\epsilon'(m)\right]^{(c_0-k)/c_0}$, and $P(\tilde{X}_1 > c_1) = ... = P(\tilde{X}_k > c_k) = \epsilon(m)\epsilon'(m)$, respectively. For simplicity's sake, we use $b_{1,k}$ to present $b_{1,k}(q_0)$.

Based on the definition, we have

$$b_{1,k} < \sum_{j=1}^{k} c_j$$

Thus, when $k = 2$,

$$\begin{aligned}
&P(\hat{X}_1 + \hat{X}_2 > b_{1,2})\\
=&P(\hat{X}_1 + \hat{X}_2 > b_{1,2}, \hat{X}_1 > b_{1,2} - c_2, \hat{X}_2 > b_{1,2} - c_1)\\
&+ P(\hat{X}_1 + \hat{X}_2 > b_{1,2}, \hat{X}_1 < b_{1,2} - c_2) + P(\hat{X}_1 + \hat{X}_2 > b_{1,2}, \hat{X}_2 < b_{1,2} - c_1)\\
=&P(\hat{X}_1 + \hat{X}_2 > b_{1,2}, c_1 > \hat{X}_1 > b_{1,2} - c_2) + P(\hat{X}_1 > c_1, \hat{X}_2 > b_{1,2} - c_1)\\
&+ P(\hat{X}_1 + \hat{X}_2 > b_{1,2}, \hat{X}_1 < b_{1,2} - c_2) + P(\hat{X}_1 + \hat{X}_2 > b_{1,2}, \hat{X}_2 < b_{1,2} - c_1)
\end{aligned}$$

Based on construction, the last three terms always smaller than $\epsilon(m)\epsilon'(m)(1+\delta_4(m))$ for $\delta_4(m) := \max_{i\in\Omega}\sup_{p\in\mathcal{P}_i'}\Big|P(\hat{T}_i < p)/P(\tilde{T}_i < p) - 1\Big| \to 0$, and accordingly, we have

$$\begin{aligned}
&P(\hat{X}_1+\hat{X}_2 > b_{1,2}, c_1 > \hat{X}_1 > b_{1,2}-c_2) + P(\hat{X}_1 > c_1, \hat{X}_2 > b_{1,2}-c_1)\\
\leq&[P(\hat{X}_1+\tilde{X}_2 > b_{1,2}, c_1 > \hat{X}_1 > b_{1,2}-c_2) + P(\hat{X}_1 > c_1, \tilde{X}_2 > b_{1,2}-c_1)](1+\delta_4(m))\\
\leq&[P(\hat{X}_1+\tilde{X}_2 > b_{1,2}, \hat{X}_1 > b_{1,2}-c_2, \tilde{X}_2 > b_{1,2}-c_1)](1+\delta_4(m))\\
\leq&P(\tilde{X}_1+\tilde{X}_2 > b_{1,2}, \tilde{X}_1 > b_{1,2}-c_2, \tilde{X}_2 > b_{1,2}-c_1)(1+\delta_4(m))^2
\end{aligned}$$

Based on similar arguments, we can also have

$$\begin{aligned}
&P(\hat{X}_1+\hat{X}_2 > b_{1,2}, c_1 > \hat{X}_1 > b_{1,2}-c_2) + P(\hat{X}_1 > c_1, \hat{X}_2 > b_{1,2}-c_1)\\
\geq&P(\tilde{X}_1+\tilde{X}_2 > b_{1,2}, \tilde{X}_1 > b_{1,2}-c_2, \tilde{X}_2 > b_{1,2}-c_1)(1-\delta_4(m))^2
\end{aligned}$$

Thus,

$$\sup_{q_0\geq\epsilon(m)\left[\epsilon'(m)\right]^{\frac{c_0-2}{c_0}}}\left|\frac{P(\hat{X}_1+\hat{X}_2 > b_{1,2})}{P(\tilde{X}_1+\tilde{X}_2 > b_{1,2})} - 1\right| \to 0$$

Similarly, if $\sup_{q_0\geq\epsilon(m)\left[\epsilon'(m)\right]^{\frac{c_0-k}{c_0}}}\left|\frac{P(\sum_{j=1}^k \hat{X}_j > b_{1,k})}{P(\sum_{j=1}^k \tilde{X}_j > b_{1,k})} - 1\right| \to 0$, we can have

$$\sup_{q_0\geq\epsilon(m)\left[\epsilon'(m)\right]^{\frac{c_0-k-1}{c_0}}}\left|\frac{P(\sum_{j=1}^{k+1} \hat{X}_j > b_{1,k+1})}{P(\sum_{j=1}^{k+1} \tilde{X}_j > b_{1,k+1})} - 1\right| \to 0$$

Thus, we can get (1). In addition, based on the similar arguments, we can get (2).

■

Proof [Proof of Lemma 8] (1) Let $Z_1', ..., Z_K' \overset{iid}{\sim} N(0,1)$, with $2 \leq K < M^{L-1}$. Define the set $\mathfrak{M} = \{\mathcal{M}_1 \subset \{1,...,m\} : 1 \leq |\mathcal{M}_1| \leq K-1\}$. It is suffice to show:

$$\lim_{m\to\infty}\sup_{\mathcal{M}_1\in\mathfrak{M}}\sup_{\substack{c_1\in[\beta_0,\gamma_m]\\ c_2\in[0,\gamma_m]}}\frac{\mathrm{P}(\frac{1}{\sqrt{K}}\sum_{i=1}^K Z_i' > c_2, \frac{1}{\sqrt{|\mathcal{M}_1|}}\sum_{j\in\mathcal{M}_1} Z_j' > c_1)}{\mathrm{P}(\frac{1}{\sqrt{K}}\sum_{i=1}^K Z_i' > c_2)} = 0$$

Here, $\beta_0 = \sqrt{2b(1-r_1)\log m + b(1-r_1)\log\log\log m}$, with

$$b = \frac{\frac{2M^{L-1}+1}{M^{L-1}+1} - r_1}{2(1-r_1)} \in \Big(\frac{M^{L-1}}{(M^{L-1}+1)(1-r_1)}, 1\Big).$$

For simplification, let $k_1 = |\mathcal{M}_1|$. For Z_1 and $Z_2 \overset{iid}{\sim} \mathrm{N}(0,1)$, define

$$\mathcal{D}_m = \left\{c_2 \in (0,\gamma_m) : \frac{d}{dc_2}\frac{P\big(\sqrt{\frac{k_1}{K}}Z_1 + \sqrt{\frac{K-k_1}{K}}Z_2 > c_2, Z_1 > \beta_0\big)}{P\big(\sqrt{\frac{k_1}{K}}Z_1 + \sqrt{\frac{K-k_1}{K}}Z_2 > c_2\big)} = 0\right\}$$

, then

$$\sup_{\substack{c_1\in[\beta_0,\gamma_m]\\ c_2\in[0,\gamma_m]}} \frac{P(\frac{1}{\sqrt{K}}\sum_{i=1}^K Z_i' > c_2, \frac{1}{\sqrt{|\mathcal{M}_1|}}\sum_{j\in\mathcal{M}_1} Z_j' > c_1)}{P(\frac{1}{\sqrt{K}}\sum_{i=1}^K Z_i' > c_2)}$$

$$\leq 2 \sup_{c_2\in[0,\gamma_m]} \frac{P\big(\sqrt{\frac{k_1}{K}}Z_1 + \sqrt{\frac{K-k_1}{K}}Z_2 > c_2, Z_1 > \beta_0\big)}{P\big(\sqrt{\frac{k_1}{K}}Z_1 + \sqrt{\frac{K-k_1}{K}}Z_2 > c_2\big)}$$

$$\leq 2\max\Bigg\{\max_{c_2=0 \text{ or } \gamma_m} \frac{P\big(\sqrt{\frac{k_1}{K}}Z_1 + \sqrt{\frac{K-k_1}{K}}Z_2 > c_2, Z_1 > \beta_0\big)}{P\big(\sqrt{\frac{k_1}{K}}Z_1 + \sqrt{\frac{K-k_1}{K}}Z_2 > c_2\big)},$$

$$\sup_{c_2\in\mathcal{D}_m} \frac{P\big(\sqrt{\frac{k_1}{K}}Z_1 + \sqrt{\frac{K-k_1}{K}}Z_2 > c_2, Z_1 > \beta_0\big)}{P\big(\sqrt{\frac{k_1}{K}}Z_1 + \sqrt{\frac{K-k_1}{K}}Z_2 > c_2\big)}\Bigg\}$$

(i). When $c_2 = 0$,

$$\lim_{m\to\infty} \frac{P\big(\sqrt{\frac{k_1}{K}}Z_1 + \sqrt{\frac{K-k_1}{K}}Z_2 > c_2, Z_1 > \beta_0\big)}{P\big(\sqrt{\frac{k_1}{K}}Z_1 + \sqrt{\frac{K-k_1}{K}}Z_2 > c_2\big)} = \lim_{m\to\infty} 2P\big(\sqrt{\frac{k_1}{K}}Z_1 + \sqrt{\frac{K-k_1}{K}}Z_2 > c_2, Z_1 > \beta_0\big) = 0$$

(ii). When $c_2 = \gamma_m$, $c_2/\beta_0 = \sqrt{\frac{1}{b(1-r_1)}}$,

$$\lim_{m\to\infty} \frac{P\big(\sqrt{\frac{k_1}{K}}Z_1 + \sqrt{\frac{K-k_1}{K}}Z_2 > c_2, Z_1 > \beta_0\big)}{P\big(\sqrt{\frac{k_1}{K}}Z_1 + \sqrt{\frac{K-k_1}{K}}Z_2 > c_2\big)} = \lim_{\beta_0\to\infty} \frac{\int_{\beta_0}^\infty \int_{S\sqrt{\frac{K}{K-k_1}}\beta_0 - \sqrt{\frac{k_1}{K-k_1}}z_1}^\infty \phi(z_1)\phi(z_2)dz_2dz_1}{\int_{S\beta_0}^\infty \phi(z)dz}$$

$$\leq C\lim_{\beta_0\to\infty} \frac{\int_{S\sqrt{\frac{K}{K-k_1}}\beta_0 - \sqrt{\frac{k_1}{K-k_1}}\beta_0}^\infty \phi(\beta_0)\phi(z)dz + \int_{\beta_0}^\infty \phi(z)\phi(S\sqrt{\frac{K}{K-k_1}}\beta_0 - \sqrt{\frac{k_1}{K-k_1}}z)dz}{\phi(S\beta_0)} \text{ (L'Hopital's rule)}$$

$$\leq C\lim_{\beta_0\to\infty} \Bigg[\exp\Big\{-\frac{\beta_0^2}{2}\Big(S\sqrt{\frac{k_1}{K-k_1}} - \sqrt{\frac{K}{K-k_1}}\Big)^2\Big\} + \int_{\beta_0}^\infty \exp\Big\{-\frac{1}{2}\Big(\sqrt{\frac{K}{K-k_1}}z - S\sqrt{\frac{k_1}{K-k_1}}\beta_0\Big)^2\Big\}dz\Bigg] = 0,$$

Where $S = \sqrt{\frac{1}{b(1-r_1)}}$

(iii). When $c_2 \in \mathcal{D}_m$, given

$$0 = \frac{d}{dc_2}\frac{P\big(\sqrt{\frac{k_1}{K}}Z_1 + \sqrt{\frac{K-k_1}{K}}Z_2 > c_2, Z_1 > \beta_0\big)}{P\big(\sqrt{\frac{k_1}{K}}Z_1 + \sqrt{\frac{K-k_1}{K}}Z_2 > c_2\big)}$$

$$= \frac{1}{P\big(\sqrt{\frac{k_1}{K}}Z_1 + \sqrt{\frac{K-k_1}{K}}Z_2 > c_2\big)^2}\times$$

$$\Big\{P\big(\sqrt{\frac{k_1}{K}}Z_1 + \sqrt{\frac{K-k_1}{K}}Z_2 > c_2\big)\frac{d}{dc_2}P\big(\sqrt{\frac{k_1}{K}}Z_1 + \sqrt{\frac{K-k_1}{K}}Z_2 > c_2, Z_1 > \beta_0\big)$$

$$- P\big(\sqrt{\frac{k_1}{K}}Z_1 + \sqrt{\frac{K-k_1}{K}}Z_2 > c_2, Z_1 > \beta_0\big)\frac{d}{dc_2}P\big(\sqrt{\frac{k_1}{K}}Z_1 + \sqrt{\frac{K-k_1}{K}}Z_2 > c_2\big)\Big\}$$

We have

$$\frac{P\left(\sqrt{\frac{k_1}{K}}Z_1+\sqrt{\frac{K-k_1}{K}}Z_2>c_2,Z_1>\beta_0\right)}{P\left(\sqrt{\frac{k_1}{K}}Z_1+\sqrt{\frac{K-k_1}{K}}Z_2>c_2\right)}=\frac{\frac{d}{dc_2}P\left(\sqrt{\frac{k_1}{K}}Z_1+\sqrt{\frac{K-k_1}{K}}Z_2>c_2,Z_1>\beta_0\right)}{\frac{d}{dc_2}P\left(\sqrt{\frac{k_1}{K}}Z_1+\sqrt{\frac{K-k_1}{K}}Z_2>c_2\right)}$$

Therefore,

$$\begin{aligned}
&\sup_{c_2\in\mathcal{D}_m}\frac{P\left(\sqrt{\frac{k_1}{K}}Z_1+\sqrt{\frac{K-k_1}{K}}Z_2>c_2,Z_1>\beta_0\right)}{P\left(\sqrt{\frac{k_1}{K}}Z_1+\sqrt{\frac{K-k_1}{K}}Z_2>c_2\right)}\\
=&\sup_{c_2\in\mathcal{D}_m}\frac{\frac{d}{dc_2}P\left(\sqrt{\frac{k_1}{K}}Z_1+\sqrt{\frac{K-k_1}{K}}Z_2>c_2,Z_1>\beta_0\right)}{\frac{d}{dc_2}P\left(\sqrt{\frac{k_1}{K}}Z_1+\sqrt{\frac{K-k_1}{K}}Z_2>c_2\right)}\\
=&\sup_{c_2\in\mathcal{D}_m}C\int_{\beta_0}^{\infty}\exp\left\{-\frac{1}{2}\left(\sqrt{\frac{K}{K-k_1}}z-\sqrt{\frac{k_1}{K-k_1}}c_2\right)^2\right\}dz\\
\le&C\int_{\beta_0}^{\infty}\exp\left\{-\frac{1}{2}\left(\sqrt{\frac{K}{K-k_1}}z-\sqrt{\frac{k_1}{K-k_1}}\gamma_m\right)^2\right\}dz\\
\to&0
\end{aligned}$$

Combine (i), (ii) and (iii), we have

$$\lim_{m\to\infty}\sup_{\mathcal{M}_1\in\mathfrak{M}}\sup_{\substack{c_1\in[\beta_0,\gamma_m]\\c_2\in[0,\gamma_m]}}\frac{P(\frac{1}{\sqrt{K}}\sum_{i=1}^K Z_i>c_2|\frac{1}{\sqrt{|\mathcal{M}_1|}}\sum_{j\in\mathcal{M}_1}Z_j|>c_1)}{P(\frac{1}{\sqrt{K}}\sum_{i=1}^K Z_i>c_2)}=0$$

(2)

It is suffice to show

$$\lim_{m\to\infty}\sup_{\mathcal{M}_1\in\mathfrak{M}}\sup_{c_2\in[0,\bar{\Phi}^{-1}(1/m)]}\frac{\mathrm{P}(\frac{1}{\sqrt{K}}\sum_{i=1}^K X_i>c_2,\frac{1}{\sqrt{|\mathcal{M}_1|}}\sum_{j\in\mathcal{M}_1}X_j>\beta_0)}{\mathrm{P}(\sum_{i=1}^K Z_i'/\sqrt{K}>c_2)}\le 0$$

Let $\breve{X}_1=\sum_{i\in\mathcal{M}_1}X_i/\sqrt{k_1}$, $\breve{X}_2=\sum_{i\in\mathfrak{M}\setminus\mathcal{M}_1}X_i/\sqrt{K-k_1}$.

Based on lemma 7, $\delta_{6m}=|P(\breve{X}_j>p)/P(Z_j>p)-1|\to 0$ uniformly for $j=1,2$ and $p>\alpha_m$.

Thus, uniformly,

$$
\begin{aligned}
&P(\sqrt{\frac{k_1}{K}}\breve{X}_1 + \sqrt{\frac{K-k_1}{K}}\breve{X}_2 > c_2, \breve{X}_1 > \beta_0)\\
=&P(\sqrt{\frac{K-k_1}{K}}\breve{X}_2 > c_2 - \sqrt{\frac{k_1}{K}}\beta_0, \breve{X}_1 > \beta_0)\\
&+ P(\sqrt{\frac{K-k_1}{K}}\breve{X}_2 < c_2 - \sqrt{\frac{k_1}{K}}\beta_0, \sqrt{\frac{k_1}{K}}\breve{X}_1 + \sqrt{\frac{K-k_1}{K}}\breve{X}_2 > c_2)\\
\leq&(1+\delta_{6m})\big[P(\sqrt{\frac{K-k_1}{K}}\breve{X}_2 > c_2 - \sqrt{\frac{k_1}{K}}\beta_0, Z_1 > \beta_0)\\
&+ P(\sqrt{\frac{K-k_1}{K}}\breve{X}_2 < c_2 - \sqrt{\frac{k_1}{K}}\beta_0, \sqrt{\frac{k_1}{K}}Z_1 + \sqrt{\frac{K-k_1}{K}}\breve{X}_2 > c_2) + P(Z_1 > \bar{\Phi}^{-1}(\alpha_m))\big]\\
\leq&(1+\delta_{6m})^2\big[P(\sqrt{\frac{k_1}{K}}Z_1 + \sqrt{\frac{K-k_1}{K}}Z_2 > c_2, Z_1 > \beta_0)\big] + (1+\delta_{6m})\sum_{j=1}^{2} P(Z_j > \bar{\Phi}^{-1}(\alpha_m))\\
\leq&(1+\delta_{6m})^2\big[P(\sqrt{\frac{k_1}{K}}Z_1 + \sqrt{\frac{K-k_1}{K}}Z_2 > c_2, Z_1 > \beta_0)\big] + 2(1+\delta_{6m})\alpha_m\\
\leq&o(\mathrm{P}(\sum_{i=1}^{K} Z_i'/\sqrt{K} > c_2))
\end{aligned}
$$

■

Proof [Proof of Lemma 9] **(i) Prove that (1) can leads to (2):**

On $\cap_{t=1}^{\ell}\mathcal{X}^{(t)}$,

$$\sum_{S\in\mathcal{B}_0^{(\ell)}} |S| I(T_S < \hat{t}^{(\ell)}) \leq \sum_{S\in\mathcal{B}_0^{(\ell)}} |S|\hat{t}^{(\ell)} + \Big\{\sum_{S\in\mathcal{B}_0^{(\ell)}} |S|\hat{t}^{(\ell)}\Big\}\epsilon$$

Combined with

$$\sum_{S\in\mathcal{B}_0^{(\ell)}} |S|\hat{t}^{(\ell)} \leq \alpha \sum_{S\in\mathcal{B}^{(\ell)}} |S|\mathbb{I}\{T_S < \hat{t}^{(\ell)}\}$$

and

$$
\begin{aligned}
\sum_{S\in\mathcal{B}^{(\ell)}} |S|\mathbb{I}\{T_S < \hat{t}^{(\ell)}\} &= \sum_{S\in\mathcal{B}_0^{(\ell)}} |S|\mathbb{I}\{T_S < \hat{t}^{(\ell)}\} + \sum_{S\in\mathcal{B}_1^{(\ell)}} |S|\mathbb{I}\{T_S^{(\ell)} \leq \hat{t}^{(\ell)}\}\\
&\leq \sum_{S\in\mathcal{B}_0^{(\ell)}} |S|\mathbb{I}\{T_S^{(\ell)} < \hat{t}^{(\ell)}\} + Cm^{r_1}
\end{aligned}
$$

We have:

$$(1-\alpha-\alpha\epsilon)\sum_{S\in\mathcal{B}_0^{(\ell)}} |S|\hat{t}^{(\ell)} \leq \alpha C m^{r_1}$$

Thus, $2|\mathcal{B}_0^{(\ell)}|\hat{t}^{(\ell)} \leq \sum_{S\in\mathcal{B}_0^{(\ell)}} |S|\hat{t}^{(\ell)} \leq \frac{\alpha}{1-\alpha-\alpha\epsilon} m^{r_1}$, for any $1 \leq \ell \leq L$.

When $\ell = 1$, by $|\mathcal{B}_0^{(1)}| = m_0 = m(1+o(1))$, we have $\hat{t}^{(\ell)} \leq Cm^{(r_1-1)}$.

When $\ell \geq 2$, on $\cap_{k=1}^{(\ell)} \mathcal{X}^{(k)}$, we have

$$\max_{k=1,\dots,\ell} \{FDP^{(k)} - \alpha\} < \epsilon$$

which leads to $|\mathcal{B}_0^{(\ell)}|/|\mathcal{B}^{(\ell)}| \to 1$. And accordingly, $\hat{t}^{(\ell)} \leq Cm^{(r_1-1)}$.

(ii) Prove that statement (2) leads to statement (3)

On layer 1, $\bar{\Phi}(\hat{c}_S) = \hat{t}^{(1)} \leq C(m)^{r_1-1}$. On layer $\ell \geq 2$ and $\cap_{h=1}^{\ell} \mathcal{X}^{(h)}$, for all $S \in \mathcal{B}^{(\ell)}$,

$$\bar{\Phi}(\hat{c}_S) \leq G_S(\hat{c}_S) + \sum_{S' \in \mathcal{U}(S)} \bar{\Phi}(\hat{c}_{S'}) \tag{S6}$$

Suppose $\bar{\Phi}(\hat{c}_{S'}) \leq C(m)^{r_1-1}$ for $S' \in \cup_{k=1}^{\ell-1} \mathcal{B}^{(k)}$, then together with $G_S(\hat{c}_S) = \hat{t}^{(\ell)} \leq Cm^{r_1-1}$ and (S6), we have

$$\hat{c}_S \geq \sqrt{2(1-r_1)\log m - 2\log\log m} = \beta_m$$

for all $S \in \mathcal{B}^{(\ell)}$.

In addition, for $S \in \mathcal{B}^{(\ell)}$, on $\cap_{h=1}^{\ell-1} \mathcal{X}^{(h)}$,

$$G_S(\hat{c}_S)[1 - \bar{\Phi}(\frac{\beta_0}{\sqrt{M^{L-1}}})]^{M^{L-1}} \leq \bar{\Phi}(\hat{c}_S) \tag{S7}$$

So we have $\bar{\Phi}(\hat{c}_S) \geq \hat{t}^{(\ell)}(1+o(1))$, and accordingly, $\hat{c}_S \leq \gamma_m$.

Note that the $\hat{c}_S \leq \gamma_m$ only depends on the statement (2) on layer $\ell - 1$. Thus, we can apply the conclusion to show $\mathrm{P}(m_0 \hat{t}^{(\ell)} > c \log m) \to 1$ in the proof of theorem 1.

(iii) Prove that statement (1) holds on layer 1 ($\ell = 1$):

Define $\nu_m = [(|\mathcal{A}'|^2/m + \delta_{2m}) \vee 1]/\sqrt{c_{\mathrm{md}} \log m}$. Let $0 = c_0 < \dots < c_{\lceil \gamma_m/\nu_m \rceil} = \gamma_m$ satisfy $c_k - c_{k-1} = \nu_m$ for $1 \leq k < \lceil \gamma_m/\nu_m \rceil$ and $c_{\lceil \gamma_m/\nu_m \rceil} - c_{\lceil \gamma_m/\nu_m \rceil - 1} \leq \nu_m$. We can get the corresponding p-values sequence $q_0 > \dots > q_{\lceil \gamma_m/\nu_m \rceil}$ with $q_k = 1 - \Phi(c_k)$. Let value $q^{(1)} = C^{(1)} c_{\mathrm{md}}/m$, by (S4), we have $P(\hat{t} > q^{(1)}) \to 1$. We define the working p-value sequence on layer 1 as $P_{sub}^{(1)} = \{q_0, \dots, q_{k^{(1)}}, q^{(1)}\}$, where $k^{(1)} \in \{0, \dots, \lceil \gamma_m/\nu_m \rceil - 1\}$ is the index s.t. $q_{k^{(1)}} \geq q^{(1)}$ and $q_{k^{(1)}+1} \leq q^{(1)}$.

If $\forall \epsilon > 0$,

$$P\left(\max_{q \in P_{sub}^{(1)}} \left| \frac{\sum_{S \in \mathcal{B}_0^{(1)}} I(X_S > \bar{\Phi}^{-1}(q)) - \sum_{S \in \mathcal{B}_0^{(1)}} P(X_S > \bar{\Phi}^{-1}(q))(1 - \delta_{0m})}{\sum_{S \in \mathcal{B}_0^{(1)}} q} \right| > \epsilon \right) \to 0 \tag{S8}$$

Then,

$$
\begin{aligned}
&P\Big(\max_{q\in P_{sub}^{(1)}}\frac{\sum_{S\in\mathcal{B}_0^{(1)}}I(T_S^{(1)}<q)-\sum_{S\in\mathcal{B}_0^{(1)}}q}{\sum_{S\in\mathcal{B}_0^{(1)}}q}>\epsilon\Big)\\
\leq&P\Big(\max_{q\in P_{sub}^{(1)}}\frac{\sum_{S\in\mathcal{B}_0^{(1)}}I(X_S>\bar\Phi^{-1}(q))-\sum_{S\in\mathcal{B}_0^{(1)}}P(\tilde X_S>\bar\Phi^{-1}(q))}{\sum_{S\in\mathcal{B}_0^{(1)}}q}>\epsilon\Big)\\
\leq&P\Big(\max_{q\in P_{sub}^{(1)}}\frac{\sum_{S\in\mathcal{B}_0^{(1)}}I(X_S>\bar\Phi^{-1}(q))-\sum_{S\in\mathcal{B}_0^{(1)}}P(X_S>\bar\Phi^{-1}(q))(1-\delta_{0m})}{\sum_{S\in\mathcal{B}_0^{(1)}}q}>\epsilon\Big)\\
\leq&P\Big(\max_{q\in P_{sub}^{(1)}}\Big|\frac{\sum_{S\in\mathcal{B}_0^{(1)}}I(X_S>\bar\Phi^{-1}(q))-\sum_{S\in\mathcal{B}_0^{(1)}}P(X_S>\bar\Phi^{-1}(q))(1-\delta_{0m})}{\sum_{S\in\mathcal{B}_0^{(1)}}q}\Big|>\epsilon\Big)\\
=&o(1) \qquad\qquad (S9)
\end{aligned}
$$

Together with the fact that $\sup_{j=1,\ldots,k}\Big|q_{(j)}/q_{(j-1)}-1\Big|=o(1)$, we have

$$
P\Big(\sup_{q\in[q^{(1)},\alpha]}\frac{\sum_{S\in\mathcal{B}_0^{(1)}}I(T_S<q)-\sum_{S\in\mathcal{B}_0^{(1)}}q}{\sum_{S\in\mathcal{B}_0^{(1)}}q}>\epsilon\Big)=o(1)
$$

Thus, to prove (1) holds on layer 1, we only need to show (S8).

Define $C_{sub}^{(1)}=\{c_0,...,c_{k'},c'\}$, with $c'=\bar\Phi^{-1}(q')$. In order to show (S8), it is suffice to show

$$
\int_0^{c'}P\Big\{\Big|\frac{\sum_{S\in\mathcal{B}_0^{(1)}}I(X_S>c)-P(X_S>c)(1-\delta_{0m})}{\sum_{S\in\mathcal{B}_0^{(1)}}\bar\Phi(c)}\Big|\geq\epsilon\Big\}dc=o(\nu_m) \qquad (S10)
$$

Note that by Markov inequality,

$$
\begin{aligned}
&P\Big\{\Big|\frac{\sum_{S\in\mathcal{B}_0^{(1)}}\big[I(X_S>c)-P(X_S>c)(1-\delta_{0m})\big]}{\sum_{S\in\mathcal{B}_0^{(1)}}\bar\Phi(c)}\Big|\geq\epsilon\Big\}\\
\leq&P\Big\{\Big|\frac{\sum_{S\in\mathcal{B}_0^{(1)}}\big[I(X_S>c)-P(X_S>c)\big]}{\sum_{S\in\mathcal{B}_0^{(1)}}\bar\Phi(c)}\Big|\geq\epsilon-(1+\delta_{0m})\delta_{0m}\Big\}\\
\leq&\frac{\sum_{S,S'\in\mathcal{B}_0^{(1)}}\big[P(X_S>c,X_{S'}>c)-P(X_S>c)P(X_{S'}>c)\big]}{\big(\sum_{S\in\mathcal{B}_0^{(1)}}\bar\Phi(c)\big)^2[\epsilon-(1+\delta_{0m})\delta_{0m}]^2}
\end{aligned}
$$

We can divide the $S,S'\in\mathcal{B}_0^{(1)}$ into the following three subsets:

$$
\begin{aligned}
\mathcal{B}_{01}^{(1)}&=\{S,S'\in\mathcal{B}_0^{(1)}:S=S'\}\\
\mathcal{B}_{02}^{(1)}&=\{S,S'\in\mathcal{B}_0^{(\ell)}:S\neq S',\exists A,A'\in\mathcal{A}^{(L)},s.t. S\subset A,S'\subset A',\text{ and }A'\in\Gamma_A\} \qquad (S11)\\
\mathcal{B}_{03}^{(1)}&=\{S,S'\in\mathcal{B}_0^{(1)}:S\neq S'\}\setminus\mathcal{B}_{02}^{(1)}
\end{aligned}
$$

Then,

$$\frac{\sum_{(S,S')\in\mathcal{B}_{01}^{(1)}}\big[P(X_S>c,X_{S'}>c)-P(X_S>c)P(X_{S'}>c)\big]}{\big(\sum_{S\in\mathcal{B}_0^{(1)}}\bar\Phi(c)\big)^2[\epsilon-(1+\delta_{0m})\delta_{0m}]^2}\leq\frac{C}{\sum_{S\in\mathcal{B}_0^{(1)}}\bar\Phi(c)}$$

Based on condition 3,

$$\frac{\sum_{(S,S')\in\mathcal{B}_{02}^{(1)}}\big[P(X_S>c,X_{S'}>c)-P(X_S>c)P(X_{S'}>c)\big]}{\big(\sum_{S\in\mathcal{B}_0^{(1)}}\bar\Phi(c)\big)^2[\epsilon-(1+\delta_{0m})\delta_{0m}]^2}\leq\frac{C(|\mathcal{A}'|^2/m+\delta_{2m})}{\sum_{S\in\mathcal{B}_0^{(1)}}\bar\Phi(c)}$$

In addition,

$$\frac{\sum_{(S,S')\in\mathcal{B}_{03}^{(1)}}\big[P(X_S>c,X_{S'}>c)-P(X_S>c)P(X_{S'}>c)\big]}{\big(\sum_{S\in\mathcal{B}_0^{(1)}}\bar\Phi(c)\big)^2[\epsilon-(1+\delta_{0m})\delta_{0m}]^2}=o(1)$$

Thus, after some calculation, we can prove (S10) and then $\mathrm{P}(\mathcal{X}^{(1)})\to 1$.

Similarly, if $|\tilde\Omega_0|=m(1+o(1))$, based on (S8), we have

$$P\bigg(\max_{q\in P_{sub}^{(1)}}\bigg|\frac{\sum_{S\in\mathcal{B}_0^{(1)}}I(T_S^{(1)}<q)-\sum_{S\in\mathcal{B}_0^{(1)}}q}{\sum_{S\in\mathcal{B}_0^{(1)}}q}\bigg|>\epsilon\bigg)=o(1)$$

Hence, $\mathrm{P}(\mathcal{X}'^{(1)})\to 1$.

(iv) Prove that statement (1) holds on layer $\ell\geq 2$ when statement (1) holds on previous layers:

On layer ℓ, we can divide the $S,S'\in\mathcal{B}_0^{(\ell)}$ into the following three subsets:

$$\mathcal{B}_{01}^{(\ell)}=\{S,S'\in\mathcal{B}_0^{(\ell)}:S=S',\{T_i:i\in S\}\text{ are mutually independent}\}$$
$$\mathcal{B}_{02}^{(\ell)}=\{S,S'\in\mathcal{B}_0^{(\ell)}:\exists A,A'\in\mathcal{A}^{(L)},s.t.S\subset A,S'\subset A',\text{ and }A'\in\Gamma_A\}$$
$$\mathcal{B}_{03}^{(\ell)}=\{S,S'\in\mathcal{B}_0^{(\ell)}:S\neq S'\}\setminus\mathcal{B}_{02}^{(\ell)}$$

Consider the p-values sequence $q_0>...>q_{\lceil\gamma_m/\nu_m\rceil}$ constructed in (iii). Let $q^{(\ell)}=C^{(\ell)}c_{\mathrm{md}}/m$, by (S4), we have $P(\hat t>q^{(\ell)})\to 1$. We define the working p-value sequence on layer 1 as $P_{sub}^{(\ell)}=\{q_0,...,q_{k^{(\ell)}},q^{(\ell)}\}$, where $k^{(\ell)}\in\{0,...,\lceil\gamma_m/\nu_m\rceil-1\}$ is the index s.t. $q_{k^{(\ell)}}\geq q^{(\ell)}$ and $q_{k^{(\ell)}+1}\leq q^{(\ell)}$.

In view of statement (3) and Lemma 8, we have

$$\sup_{k=0,...,\lceil\gamma_m/\nu_m\rceil}\bigg|\frac{G_S(c_k)}{\bar\Phi(c_k)}-1\bigg|=o(1)$$

Together with statement (3) and Lemma 7, there exists $\delta_5(m)\to 0$ with

$$\begin{aligned}&\max_{S\in\mathcal{B}_0^{(\ell)}}\frac{\mathrm{P}(X_S>\bar\Phi^{-1}(q)|\mathcal{Q}^{(1:\ell-1)})}{q}\\&\leq\max_{S\in\mathcal{B}_0^{(\ell)}}\frac{P(X_S>\bar\Phi^{-1}(q))}{P(Z_S>\bar\Phi^{-1}(q))[1-\bar\Phi(\frac{\beta_0}{\sqrt{M^{L-1}}})]^{M^{L-1}}}\\&\leq 1+\delta_5(m)\end{aligned}$$

Then $\forall \epsilon > 0$, by following the similar arguments in (iii), we can have

$$P\left(\max_{q \in P_{sub}^{(\ell)}} \left| \frac{\sum_{S \in \mathcal{B}_{01}^{(\ell)}} |S| I(X_S > \bar{\Phi}^{-1}(q)) - \sum_{S \in \mathcal{B}_{01}^{(\ell)}} |S| P(X_S > \bar{\Phi}^{-1}(q) | \mathcal{Q}^{(1:\ell-1)})(1+\delta_{0m})}{\sum_{S \in \mathcal{B}_{01}^{(\ell)}} |S| q} \right| > \epsilon \middle| \mathcal{Q}^{(1:\ell-1)} \right) \rightarrow 0 \tag{S12}$$

Then,

$$\begin{aligned} & P\left(\max_{q \in P_{sub}^{(\ell)}} \frac{\sum_{S \in \mathcal{B}_0^{(\ell)}} |S| I(T_S < q) - \sum_{S \in \mathcal{B}_0^{(\ell)}} |S| q}{\sum_{S \in \mathcal{B}_0^{(\ell)}} |S| q} > \epsilon \middle| \mathcal{Q}^{(1:\ell-1)} \right) \\ \leq & P\left(\max_{q \in P_{sub}^{(\ell)}} \frac{\sum_{S \in \mathcal{B}_0^{(\ell)}} |S| I(X_S > \bar{\Phi}^{-1}(q)) - \sum_{S \in \mathcal{B}_0^{(\ell)}} |S| P(X_S > \bar{\Phi}^{-1}(q) | \mathcal{Q}^{(1:\ell-1)})}{\sum_{S \in \mathcal{B}_0^{(\ell)}} |S| q} > \epsilon/2 \middle| \mathcal{Q}^{(1:\ell-1)} \right) \\ = & o(1) \end{aligned} \tag{S13}$$

Together with the fact that $\sup_{j=1,\dots,k} \left| q_{(j)}/q_{(j-1)} - 1 \right| = o(1)$, we have

$$P\left(\sup_{q \in [q^{(\ell)}, \alpha]} \frac{\sum_{S \in \mathcal{B}_0^{(\ell)}} |S| I(T_S < q) - \sum_{S \in \mathcal{B}_0^{(\ell)}} |S| q}{\sum_{S \in \mathcal{B}_0^{(\ell)}} |S| q} > \epsilon \middle| \mathcal{Q}^{(1:\ell-1)} \right) = o(1)$$

And thus $P(\mathcal{X}^{(\ell)}) \rightarrow 1$.

Similarly, when $|\tilde{\Omega}_0| = m(1 + o(1))$, we have $P(\mathcal{X}'^{(\ell)}) \rightarrow 1$ based on Lemma 8 (2). ■

Proof [Proof of Lemma 10] When $\ell = 1$:

for $\delta = 1/m^4$,

$$\begin{aligned} \sum_{S \in \mathcal{B}_0^{(1)}} |S| \hat{t}^{(1)} & \leq \alpha \sum_{S \in \mathcal{B}^{(1)}} |S| I(T_S < \hat{t}^{(1)}) \\ & \leq \alpha \sum_{S \in \mathcal{B}^{(1)}} |S| I(T_S < \hat{t}^{(1)} + \delta) \\ & \leq \sum_{S \in \mathcal{B}_0^{(1)}} |S| \hat{t}^{(1)} (1 + o(1)) \end{aligned} \tag{S14}$$

Assume (12) holds on layer $1, \dots, \ell - 1$. Then,

$$\sum_{S \in \mathcal{B}_0^{(\ell)}} |S| \hat{t}^{(\ell)} \leq \alpha(1 + o(1)) \sum_{S \in \mathcal{B}^{(\ell)}} |S| I(T_S < \hat{t}^{(\ell)})$$

Thus, by following the similar arguments on (S14), we can get (12) on layer ℓ.

■
